# Characterisation of three fungal glucuronoyl esterases on glucuronic acid ester model compounds

**DOI:** 10.1007/s00253-017-8266-9

**Published:** 2017-04-20

**Authors:** Silvia Hüttner, Sylvia Klaubauf, Ronald P. de Vries, Lisbeth Olsson

**Affiliations:** 1 Division of Industrial Biotechnology, Department of Biology and Biological Engineering, Chalmers University of Technology, SE-412 96 Gothenburg, Sweden; 2 Wallenberg Wood Science Center, Chalmers University of Technology, SE-412 96 Gothenburg, Sweden; 3 Fungal Physiology, CBS-KNAW Fungal Biodiversity Centre & Fungal Molecular Physiology, Utrecht University, 3584 CT Utrecht, The Netherlands

**Keywords:** Carbohydrate esterase, Benzyl glucuronic acid, Allyl glucuronic acid, Methyl glucuronic acid, Lignin-carbohydrate complexes, CAZymes

## Abstract

The glucuronoyl esterases (GEs) that have been identified so far belong to family 15 of the carbohydrate esterases in the CAZy classification system and are presumed to target ester bonds between lignin alcohols and (4-O-methyl-)d-glucuronic acid residues of xylan. Few GEs have been cloned, expressed and characterised to date. Characterisation has been done on a variety of synthetic substrates; however, the number of commercially available substrates is very limited. We identified novel putative GEs from a wide taxonomic range of fungi and expressed the enzymes originating from *Acremonium alcalophilum* and *Wolfiporia cocos* as well as the previously described PcGE1 from *Phanerochaete chrysosporium*. All three fungal GEs were active on the commercially available compounds benzyl glucuronic acid (BnGlcA), allyl glucuronic acid (allylGlcA) and to a lower degree on methyl glucuronic acid (MeGlcA). The enzymes showed pH stability over a wide pH range and tolerated 6-h incubations of up to 50 °C. Kinetic parameters were determined for BnGlcA. This study shows the suitability of the commercially available model compounds BnGlcA, MeGlcA and allylGlcA in GE activity screening and characterisation experiments. We enriched the spectrum of characterised GEs with two new members of a relatively young enzyme family. Due to its biotechnological significance, this family deserves to be more extensively studied. The presented enzymes are promising candidates as auxiliary enzymes to improve saccharification of plant biomass.

**Electronic supplementary material:**

The online version of this article (doi:10.1007/s00253-017-8266-9) contains supplementary material, which is available to authorized users.

## Introduction

Glucuronoyl esterases (GEs) are members of the carbohydrate esterase 15 family (CE15) in the CAZy classification (www.cazy.org) (Li et al. 2007; Lombard et al. 2014). This class of enzymes was discovered in 2006 and is believed, in nature, to hydrolyse ester bonds between aliphatic alcohols in lignin and the 4-O-methyl-d-glucuronic acid side chains of xylan (Špániková and Biely 2006). CE15 representatives are predicted in the genomes of a wide range of fungi and bacteria. To date, 11 GEs have been described (for a list, see Table S1). Several GEs that have been reported in the literature are bimodular and consist of a catalytic domain, a linker region and an N-terminal family 1 carbohydrate-binding module (CBM). The presumed major function of the CBM is to enhance enzymatic activity by binding to insoluble substrates (Várnai et al. 2014).

**Fig. 1:**
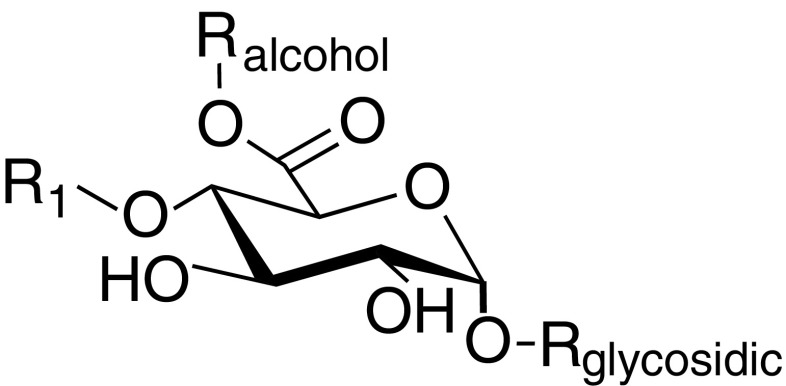
Generalised structure of synthetic GE substrates. R_1_ = H, CH_3_; R_alcohol_ = methyl, aryl, lignin structures; R_glycosidic_ = H, d-xylopyranoside. In plant cell walls, lignin and hemicelluloses are cross-linked by (4-O-methyl-)d-glucuronic acid residues linked to glucuronoxylan (R_glycosidic_) and ester bonds to hydroxyl groups of lignin (R_alcohol_)

GEs have been characterised on a variety of synthetic substrates that mimic the structure of lignin-carbohydrate complexes (LCCs) (Biely et al. 2015; D’Errico et al. 2015, 2016; Nylander et al. 2015; Ďuranová et al. 2009a, b; Katsimpouras et al. 2014; Li et al. 2007). Variations of the overall structure of these substrates (Fig. 1) include methyl and simple aryl structures as well as lignin dimers at the alcohol position (R_alcohol_ in Fig. 1). In the aryl esters, R_1_ and R_glycosidic_ have so far been limited to hydrogens and methyl groups. In addition, structures with methyl esters of 4-O-methyl-d-glucuronic acid α1-2-linked to xylose units have been used (Špániková et al. 2007). Of the many synthetic substrates used for GE assays, only a few are commercially available; benzyl-d-glucuronic acid (BnGlcA), allyl-d-glucuronic acid (allylGlcA) and methyl-d-glucuronic acid (MeGlcA). Of those, the d-benzyl glucuronic acid ester (R_alcohol_ = benzyl; R_1_, R_glycosidic_ = H) was reported to be suitable for quantitative estimation of GE activity using HPLC, while allylGlcA (R_alcohol_ = allyl; R_1_, R_glycosidic_ = H) and MeGlcA (R_alcohol_ = methyl; R_1_, R_glycosidic_ = H) have only been used in TLC-based assays (Sunner et al. 2015; De Santi et al. 2016). In a recent study, Arnling Bååth et al. also demonstrated GE action on a natural substrate by using native lignin-carbohydrate ester bonds from spruce and birch (Arnling Bååth et al. 2016).

The industrial applications of GEs lie primarily in the production of biofuels and biomaterials from plant biomass, where they were shown to increase substrate hydrolysis (D’Errico et al. 2016). It was recently demonstrated that GEs can cleave the ester bond between lignin and glucuronoxylan (Arnling Bååth et al. 2016), pointing to the potential of this enzyme to aid in targeted decomposition of biomass. As GEs enable selective cleavage of alkali-labile bonds at acidic pH values, GEs would also allow biomass degradation at a lower pH where other alkali-labile structures can be retained. In addition, production of alkyl-branched glucuronic acid derivatives using the synthetic capabilities of GEs could yield bioactive compounds such as nonionic surfactants for cosmetic and pharmaceutical applications (Moreau et al. 2004) and improved prodrugs against tumours (de Graaf et al. 2004). To support such developments, substantial effort is required to broaden our knowledge about GEs and their hydrolytic and synthetic potentials.

To this end, we cloned, expressed and characterised CE15 domain-containing proteins from five fungi living in various habitats, and thus exhibiting potentially different properties. This set of fungi consists of two ascomycetes (*Acremonium alcalophilum*, *Lentithecium fluviatile*) and three basidiomycetes (*Wolfiporia cocos*, *Schizophyllum commune*, *Phanerochaete chrysosporium*). Characterisation of the enzymatic activity of these fungal enzymes was primarily performed on BnGlcA, using a recently developed spectrophotometric assay (Sunner et al. 2015). Two additional synthetic substrates, MeGlcA and allylGlcA, were used to compare the specificities of the tested enzymes.

## Materials and methods

### Gene selection and phylogeny

The protein sequences of the *Acremonium alcalophilum* v2.0, *Lentithecium fluviatile* CBS 122367 v1.0, *Schizophyllum commune* v3.0 (Ohm et al. 2010), *Phanerochaete chrysosporium* v2.0 (Martinez et al. 2004) and *Wolfiporia cocos* v1.0 (Floudas et al. 2012) genomes—all found in the publicly available JGI Genome databases at http://genome.jgi.doe.gov/—were analysed for CE15 family members, which yielded the putative GEs AaGE1, LfGE1, LfGE2 LfGE3, ScGE2, PcGE1 and WcGE1 (protein sequences in Fig. S1). Gene models were verified by BLAST against EST databases, with RNA-Seq data (if available) and multiple sequence alignment. SignalP v4.1, http://www.cbs.dtu.dk, was used to detect the presence of secretory signal peptides (Petersen et al. 2011), and domains of carbohydrate active enzymes were identified with dbCAN, http://csbl.bmb.uga.edu/dbCAN/ (Yin et al. 2012). N- and O-glycosylation sites were predicted with NetNGlyc 1.0 Server (http://www.cbs.dtu.dk/services/NetNGlyc/) and YinOYang 1.2 (http://www.cbs.dtu.dk/services/YinOYang/) (Gupta and Brunak 2002), respectively. Protein sequences were aligned using MAFFT version 7 (http://mafft.cbrc.jp/alignment/server/index.html) (Katoh and Standley 2013). Phylogenetic and molecular evolutionary analyses were conducted using MEGA version 6 (Tamura et al. 2013). Evolutionary history was inferred using the maximum likelihood method based on the Whelan and Goldman model (Whelan and Goldman 2001). A discrete gamma distribution was used to model evolutionary rate differences among sites (five categories (+G, parameter = 1.9893)). The rate variation model allowed for some sites to be evolutionarily invariable ([+I], 4.0002% sites). All positions containing gaps and missing data were eliminated.

### Cloning of putative fungal GE genes

Codon-optimised genes excluding signal peptide were synthesised (NZYTech, Portugal) and cloned into the Pichia expression vector pPICZαA (Thermo Fisher Scientific) using FastDigest *Eco*RI and FastDigest *Xba*I (Thermo Fisher Scientific) in frame with the N-terminal α-factor and the C-terminal myc- and 6XHis-tag. Briefly, DNA molecules were purified using the Illustra GFX GelBand purification kit (GE Healthcare) and vector and respective gene inserts ligated with T4 DNA ligase according to the manufacturer’s protocol (Thermo Fisher Scientific). *Escherichia coli* DH5α (Thermo Fisher Scientific) was transformed using electroporation on a Biorad MicroPulser, and clones containing the heterologous plasmid were selected on a low salt LB-medium containing 25 mg L^−1^ Zeocin (Invivogen, USA) according to the Pichia Expression Kit manual (Thermo Fisher Scientific). Plasmid DNA from transformed *E. coli* cells was isolated out using GeneJET Plasmid Miniprep Kit (Thermo Fisher Scientific), and clones were confirmed by PCR analysis and DNA sequencing (Eurofins Genomics GmbH, Germany) using AOX1 forward and reverse primers (Thermo Fisher Scientific). In addition, a WcGE1 construct without myc-tag was generated by fusion PCR: the WcGE1 CDS was PCR amplified from pPICZα-WcGE1using the primer pair pPIC_Wol8-F (5′-GAGAGGCTGAAGCTGAATTCTTGCCACCTTCCCAAG-3′; restriction site underlined) and pPIC_Wol8-R (5′-ATGGTCGACGGCGCTATTATCCAACTGAGGGGTTGTCC-3′) to generate overhangs complementary to pPICZαA. Primers were designed using Snapgene (version 2.8.1) software. PCR reactions were performed as 20 μL reactions containing 0.4 U Phusion HF polymerase (Thermo Fisher Scientific), 1× Phusion HF Buffer (Thermo Fisher Scientific), 0.2 mM dNTPs, 0.5 μM primers. The PCR conditions were: initial denaturation for 30 s at 98 °C, 10 cycles of denaturation for 10 s at 98 °C, annealing at 65 °C for 15 s and extension for 25 s at 72 °C, 20 cycles of denaturation for 10 s at 98 °C and extension for 25 s at 72 °C, followed by final extension for 7 min at 72 °C. Gel purified cut vector was PCR amplified with primer pairs MssI-F (5′-CCCCAAATGGCCCAAAACTGAC-3′) and pPIC_EcoRI-R (5′-GAATTCAGCTTCAGCCTCTC-3′), and MssI-R (5′-AACATGAAACTATTTGACCCCACACTCAG-3′) and pPIC-SalI-F (5′-AATAGCGCCGTCGACC-3′) in 30 cycles of 10 s at 98 °C, 15 s at 61 °C and 70 s at 72 °C, generating two fragments. PCR-amplified vector fragments and insert were fused in an overlap PCR reaction containing 0.4 U Phusion HF polymerase, 1× Phusion HF Buffer, 0.2 mM dNTPs and 2 μL of 3× diluted 1:1 mixture of vector and insert. The PCR conditions were as follows: initial denaturation for 30 s at 98 °C, 30 cycles of denaturation for 10 s at 98 °C, 15 s annealing at 61 °C and extension for 70 s at 72 °C, followed by final extension for 5 min at 72 °C. *E. coli* was directly transformed with overlap PCR products and transformants were examined as stated above.

### Heterologous expression of putative GEs in *Pichia pastoris*

The *Mss*I linearised pPICZαA-GE constructs were purified by Phenol:Chloroform:Isoamylalcohol (Sigma-Aldrich) extraction and ethanol precipitation according to the Pichia Expression Kit manual (Thermo Fisher Scientific). The protease-deficient *P. pastoris* strain SMD1168H (Thermo Fisher Scientific) was transformed by electroporation, and positive clones were selected on a YPDS medium using 100 mg L^−1^ Zeocin. Integration of vector constructs into the genome was confirmed by PCR screening with AOX1 primers. Expression experiments were carried out according to the Pichia Expression Kit manual (Thermo Fisher Scientific). Up to eight positive clones were screened for high protein expression in small-scale expression trials followed by activity assays. For enzyme production, induction was performed at 25 °C and 150 rpm for up to 7 days. Samples were taken once a day and the supernatants analysed for activity in the extracellular medium. A *P. pastoris* strain expressing StGE2 from *Myceliophthora thermophila* was kindly provided by the OPTIBIOCAT consortium and protein produced as described above.

### Purification of recombinant enzymes

Two different purification strategies on the Äkta system (GE Healthcare) were performed for three of the produced enzymes: immobilised metal ion affinity chromatography (IMAC) for AaGE1 and WcGE1, and ion exchange chromatography (IEX) for PcGE1. Purification via IMAC was performed on HisPrepFF 16/10 or HisTrap Excel columns (GE Healthcare) according to the manufacturer’s recommendations. Elution was performed in one step with a buffer containing 500 mM imidazole, and flow-through and elution fractions showing UV absorbance at 280 nm were buffer-exchanged and examined for GE activity. For anion exchange chromatography, a 1 mL Q-Sepharose HiTrapFF column (GE Healthcare) was used. For PcGE1 (theoretical pI of 5.7) 0.02 M Tris-HCl, pH 8.5, was chosen as loading buffer and elution was performed with loading buffer containing an increasing gradient of NaCl up to a final concentration of 1 M. Fractions of all steps showing UV absorbance at 280 nm were collected and analysed for GE activity. Purity of target enzymes was assessed using SDS-PAGE (Bio-Rad) followed by Coomassie Brilliant Blue R-250 staining or imaging on a Chemidoc Touch Stain-free Imager (Bio-Rad). Presence of N-glycosylation on purified proteins was determined by digestion with PNGase F (New England Biolabs) according to the manufacturer’s protocol.

### Protein and enzyme assays

BnGlcA, allylGlcA and MeGlcA (Carbosynth, Compton, UK) were dissolved in 100% dimethyl sulfoxide (DMSO) to a 100 mM substrate stock and stored at −20 °C. GE activity was detected by using the K-URONIC kit (Megazyme, Ireland), as described previously (Sunner et al. 2015). Briefly, GE assays were performed in 200 μL reactions containing 73 mM potassium phosphate buffer pH 6.0, 2 mM substrate and 2% DMSO, and incubated for 1–30 min at 35 °C. Heat-inactivated enzyme samples were treated the same way and served as background controls. Fifty microlitres of the reaction mix were transferred to a microplate in technical triplicates, and the concentration of released GlcA was determined in a 250 μL detection assay (K-URONIC kit) at 25 °C for 10 min in absorbance mode (FLUOstar Omega, BMG LABTECH, Germany).

To determine the optimal storage conditions, purified AaGE1, PcGE1 and WcGE1 were stored at 4 °C for 1 week in four different buffers: 0.1 M citrate-phosphate buffer, pH 3–7, 0.1 M potassium-phosphate buffer, pH 6–8, 0.1 M Tris-HCl buffer, pH 7–9 and 0.1 M glycine-NaOH buffer, pH 9–11. After incubation, the enzymes were buffer-exchanged to the BnGlcA assay buffer (0.1 M potassium-phosphate buffer, pH 6.0) using Amicon centrifugal filters with a cut-off of 10 kDa (Merck Millipore) and GE activity was determined by hydrolysis of BnGlcA. Protein concentration was determined either by Pierce BCA protein assay (Thermo Fisher Scientific) or densitometric analysis in comparison to bovine serum albumin standards.

### Nucleotide sequence accession numbers

The nucleotide sequences of the synthesised codon-optimised expression constructs are available in GenBank (KY450654, AaGE1; KY450655, LfGE1; KY450656, LfGE2; KY450657, LfGE3; KY450658, PcGE1; KY450659, ScGE2; KY450660, WcGE1). The nucleotide sequence of the original AaGE1 is available in GenBank under the accession number KX898021. The nucleotide sequence of WcGE1 is available in the Third Party Annotation Section of the DDBJ/ENA/GenBank databases under the accession number TPA: BK009982.

## Results

The growing number of available microbial genomes allows for the identification of new enzymes with potentially new characteristics. In order to identify new GEs, so far uncharacterised proteins with CE15 domains were chosen and their hydrolytic potential on three different synthetic substrates was evaluated.

### Identification, selection and annotation of putative fungal glucuronoyl esterases

**Fig. 2:**
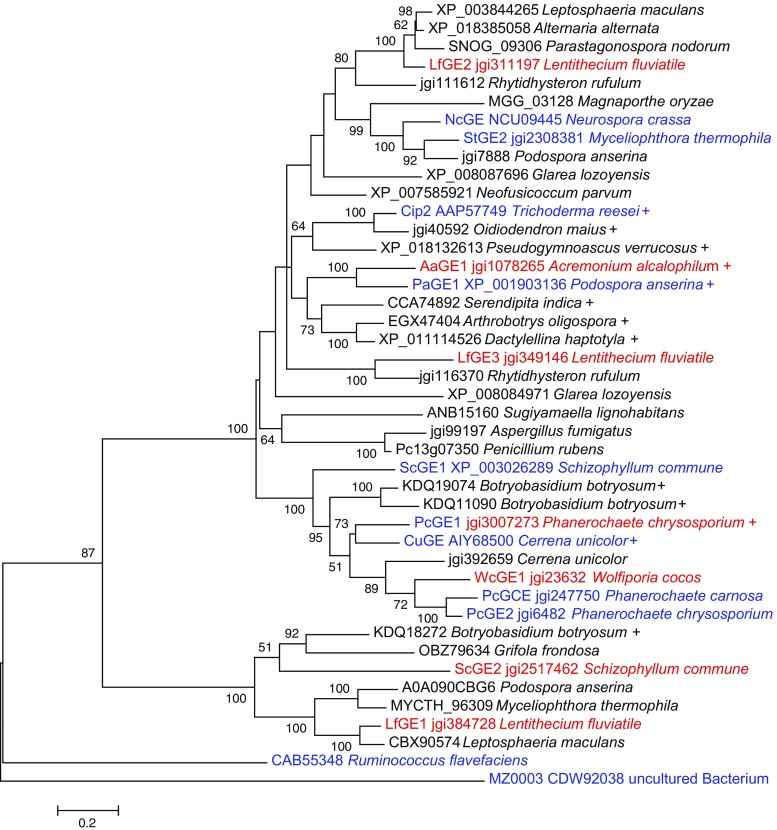
Evolutionary relationships of CE15 proteins inferred by maximum likelihood method. Characterised GEs (*text in blue*), GE candidates selected in this study (*text in red*) and presence of CBM (*plus signs*) are indicated. Bootstrap (500 replicates) values above 50% are shown next to the branches. The tree is drawn to scale, with branch lengths measured in the number of substitutions per site. For a detailed list of proteins, see Table S1 (colour figure online)

For an overview of the phylogenetic relationships of GEs, all CE15 proteins characterised to date as well as a selection of putative fungal GEs comprising a wide taxonomic range were used to construct a phylogenetic tree (Fig. 2). The phylogenetic tree, as well as ecological information about the included fungi, served as a starting point for the selection of GE candidates with potential diversity regarding their pH and temperature optima. The following seven proteins, originating from five species of *Ascomycota* and *Basidiomycota*, were selected: AaGE1 from *A. alcalophilum*; LfGE1, LfGE2 and LfGE3 from *L. fluviatile*; PcGE1 from *P. chrysosporium*; ScGE2 from *S. commune*; and WcGE1 from *W. cocos*.

*Acremonium alcalophilum* is one of only a few known alkalophilic, cellulolytic fungi that can be cultured in laboratory environments (Nagai et al. 1995; Grum-Grzhimaylo et al. 2013). No alkali-tolerant GE has been characterised to date. *Lentithecium* species are saprobic and live in freshwater habitats. Three proteins have been annotated as CE15 family members in the genome of *L. fluviatile*, and they were selected to study differentiation of CE15 family members in the same organism. *Schizophyllum commune* is a ubiquitous white-rot fungus growing on softwood, hardwood and grasses (Ohm et al. 2010). One of the two CE15 family members identified in the genome of *S. commune*, ScGE1, has already been characterised (Špániková and Biely 2006; Wong et al. 2012), and we therefore selected ScGE2. The brown-rot fungus *W. cocos*, also known as *W. extensa* or Fu Ling in Chinese medicine, parasitises the roots of trees (Milagres and Sales 2001; Shu et al. 2013). One putative GE has been annotated in the genome of *W. cocos* (Floudas et al. 2012), WcGE1. Two glucuronoyl esterases from the white-rot fungus *P. chrysosporium* have been characterised so far: PcGE1 and PcGE2 (Ďuranová et al. 2009a; Ďuranová et al. 2009b). PcGE1 was included in this study as a reference point.

A list of the selected proteins and information on their domain organisation is summarised in Table 1, while protein sequences for the respective proteins can be found in Fig. S1.

**Table 1:** Putative GEs investigated in the present study

Phylum	Organism	Protein	JGI protein ID	Protein length^a^	Signal peptide cleavage site between	CBM1 domain^b^	CE15 domain^b^
*Ascomycota*	*A. alcalophilum*	AaGE1	Acral2|1078265	496 aa	aa 19/20	aa 23–51	aa 143–463
	*L. fluviatile*	LfGE1	Lenfl1|384728	404 aa	aa 17/18	–	aa 58–373
	*L. fluviatile*	LfGE2	Lenfl1|311197	388 aa	aa 16/17	–	aa 39–359
	*L. fluviatile*	LfGE3	Lenfl1|349146	387 aa	aa 16/17	–	aa 35–356
*Basidiomycota*	*P. chrysosporium*	PcGE1	Phchr1|130517	472 aa	aa 19/20	aa 25–52	aa 111–438
	*S. commune*	ScGE2	Schco3|2517462	389 aa	aa 18/19	–	aa 38–361
	*W. cocos*	WcGE1	Wolco1|23632	408 aa	aa 19/20	–	aa 45–373

^a^Protein length including signal peptide

^b^Domains were identified by sequence-based predictions (see “Material and methods” section)

### Cloning, expression and purification of putative GEs

**Fig. 3:**
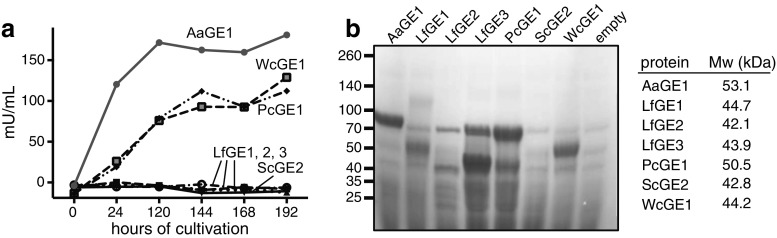
Heterologous expression of GEs in *P. pastoris*. **a** Progression of GE activity over the course of the *Pichia* cultivation. Activity on crude culture filtrates was tested on 2 mM BnGlcA at pH 6.0. **b** Crude culture filtrates of 8-day-old *Pichia* cultures were separated on SDS-PAGE and stained with Coomassie Brilliant Blue. Calculated molecular weights (Mw; in kDa) of heterologously expressed, mature proteins are listed

Production and secretion of recombinant proteins in the *P. pastoris* expression system was followed by measuring the total protein concentration in the cultivation medium as well as the GE activity by hydrolysis of BnGlcA. Over the course of an 8-day cultivation, extracellular total protein increased continuously for all tested candidates. However, only three of the seven putative CE15-domain-containing proteins showed activity on BnGlcA: AaGE1, PcGE1 and WcGE1 with 181, 112 and 129 mU mL^−1^ after 192 h, respectively (Fig. 3a).

Putative GE bands appeared for 8-day-old cultures of AaGE1, LfGE1, LfGE3, PcGE1 and WcGE1, respectively, while cultures expressing LfGE2 or ScGE2, respectively, resulted in less pronounced staining (Fig. 3b).

**Fig. 4:**
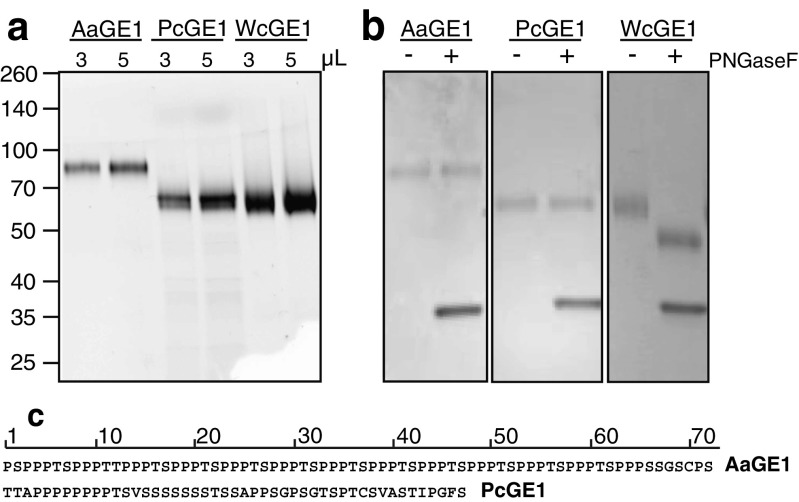
Purified GEs separated on SDS-PAGE. **a** Three GEs were purified to homogeneity and different volumes of purified protein separated on SDS-PAGE. **b** PNGase F digestion confirmed the absence of N-glycans on AaGE1 and PcGE1, whereas WcGE1 showed a clear shift. The band at 35 kDa is the PNGase F enzyme. **c** Sequences of proline-rich linker regions between CBM1 and CE15 domains in AaGE1 and PcGE1

Since no enzymatic activity could be detected for four of the candidates, even when additional clones were checked, we conducted subsequent experiments with only the three enzymes that showed activity on BnGlcA. AaGE1 and WcGE1 were purified to homogeneity from the crude *Pichia* extracts using IMAC. PcGE1 was only found in the flow-through of IMAC and was therefore purified using a Q-Sepharose column (Fig. 4a).

Calculated and observed molecular weights of proteins often differ due to posttranslational modifications of the mature protein, which can include N- and O-glycosylation, and other protein decorations, as well as disulphide bonds (Mann and Jensen 2003). Neither AaGE1 nor PcGE1 contain the Asn-Xaa-Ser/Thr sequon that usually signifies an N-glycosylation site in eukaryotes, whereas WcGE1 is predicted to contain between two and five sites (Fig. S1). As expected, bands of purified AaGE1 and PcGE1 did not show a shift after PNGase F digestion when separated on SDS-PAGE, while a shift of about 10 kDa in WcGE1 confirmed the presence of up to 5 N-glycans (Fig. 4b).

Similar to many cellulases and xylanases, for which the functional domains are separated from each other (Gilkes et al. 1991), AaGE1 contains a linker region between CBM1 and the catalytic CE15 domain (Figs. 4c and S1). CBM1s are exclusively found in fungal cellulases and hemicellulases and help the enzymes bind to the cellulose surface. Furthermore, it has been reported that CBM1s can enhance synergistic effects during hydrolysis of lignocelluloses (Inoue et al. 2015). The linker region is thought to act as a flexible arm that connects the catalytic domain to the substrate-binding module, while leaving a limited range to move freely (Schwarz 2001). The linker regions of glycoside hydrolases show low sequence conservation and vary in amino acid composition between species, but are generally very proline-, threonine- and serine-rich (Sammond et al. 2012). The 72-aa-long linker region of AaGE1 consists of 57% proline, 22% serine and 18% threonine. O-glycosylation on the 13 threonine residues in the linker likely accounts for the about 27-kDa difference in theoretical and observed molecular weight (Figs. S1 and S2).

The observed molecular weight of PcGE1 is about 10 kDa higher than calculated, which could be—as in the case of AaGE1—due to a heavily O-glycosylated linker region between the CBM1 and the CE15 catalytic domain (Figs. 4c, S1, and S2). The linker regions of AaGE1 and PcGE1 differ from each other in sequence and show only 28% overall identity. The PcGE1 linker consists of 28% proline, 34% serine and 15% threonine. The difference of only about 10 kDa between theoretical and observed molecular weight of PcGE1, compared to 27 kDa in AaGE1, is in accordance with the lower number of potentially O-glycosylated threonines.

AaGE1 was also subjected to chymotrypsin and trypsin digestions, and the peptides were identified by LC-MS/MS. Peptides covering both the N- and C-terminus were detected, confirming that the whole protein was expressed. In total, 73% of the protein sequence was covered by peptides of high statistical significance (99%). Of the remaining 139 aa that were not covered, a stretch of 90 aa included most of the proline-rich linker region, which is not cut by chymotrypsin and trypsin (Fig. S3).

### Effect of pH and temperature on enzyme stability

**Fig. 5:**
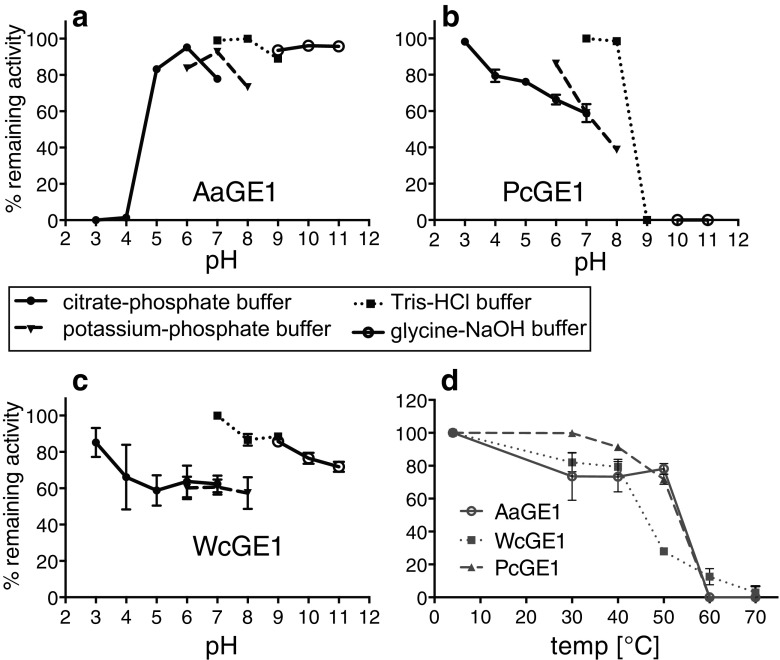
Optimal pH and temperature stability of the three GEs. **a**–**c** The pH stability was determined by incubating the enzymes in different buffers at 4 °C for 1 week, and then assessing their enzymatic activity on 2 mM BnGlcA in 0.1 M potassium-phosphate buffer, pH 6.0, at 35 °C. The enzyme activities were normalised to the highest enzyme activity. **d** Temperature stability was determined by measuring residual activity after incubation at different temperatures for 6 h. Values were normalised to the enzyme activity at 4 °C (=100%). *Error bars* (some smaller than data marker size) represent standard deviations (triplicates)

pH stability was investigated by storing the purified enzymes in buffers of different pH values at 4 °C for 1 week. AaGE1 was most stable in Tris-HCl buffer pH 8 and exhibited high stability from neutral to very alkaline pH values (Fig. 5a). In contrast, PcGE1 showed higher activity after storage in acidic to neutral pH, the optimum being Tris-HCl buffer pH 7 (Fig. 5b). Stability of WcGE1 was very buffer-dependent, the optimum storage condition was Tris-HCl buffer pH 7, the same conditions as for PcGE1 (Fig. 5c). Temperature stability was assessed by incubating the enzymes in their ideal buffers, determined by the previous experiment, for 6 h at different temperatures. All three enzymes showed a similar pattern, with a sharp decline in activity after incubation at 50 and 60 °C (Fig. 5d). AaGE1 and particularly PcGE1 were more tolerant to higher temperatures, while WcGE1 was the least stable among the three enzymes.

### Kinetic parameters and substrate specificity

**Fig. 6:**
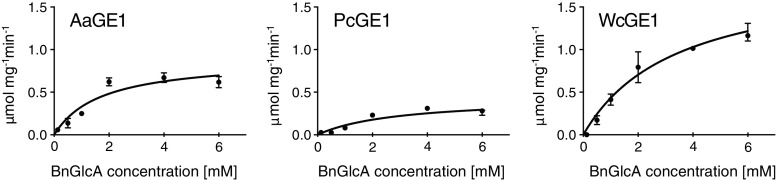
Enzyme activity on BnGlcA using AaGE1, PcGE1 and WcGE1 with the best fit to Michaelis-Menten kinetics. Values are taken from three independent measurements, and the standard deviation is represented as *error bars* (some smaller than data marker size)

Kinetic parameters of AaGE1, PcGE1 and WcGE1 were determined using BnGlcA as a substrate assayed by the standard spectrophotometric assay at pH 6.0. The *K*_*m*_ values for all three GEs were in the same order of magnitude; AaGE1 was found to have a slightly higher binding affinity (i.e. lower *K*_*m*_) than PcGE1 and WcGE1 (Table 2, Fig. 6). The highest *v*_*max*_ and *k*_*cat*_ values were reached with WcGE1.

**Table 2:** Kinetic parameters for AaGE1, PcGE1 and WcGE1 using BnGlcA as substrate

	*K*_*m*_ (mM)	*v*_*max*_ (μmol mg^−1^ min^−1^)	*k*_*cat*_ (s^−1^)
AaGE1	1.7 (±0.5)	0.90 (±0.18)	0.79
PcGE1	2.9 (±1.2)	0.44 (±0.08)	0.37
WcGE1	3.4 (±0.8)	1.91 (±0.22)	1.45

### GE activity on allyl and methyl glucuronic acid esters

**Fig. 7:**
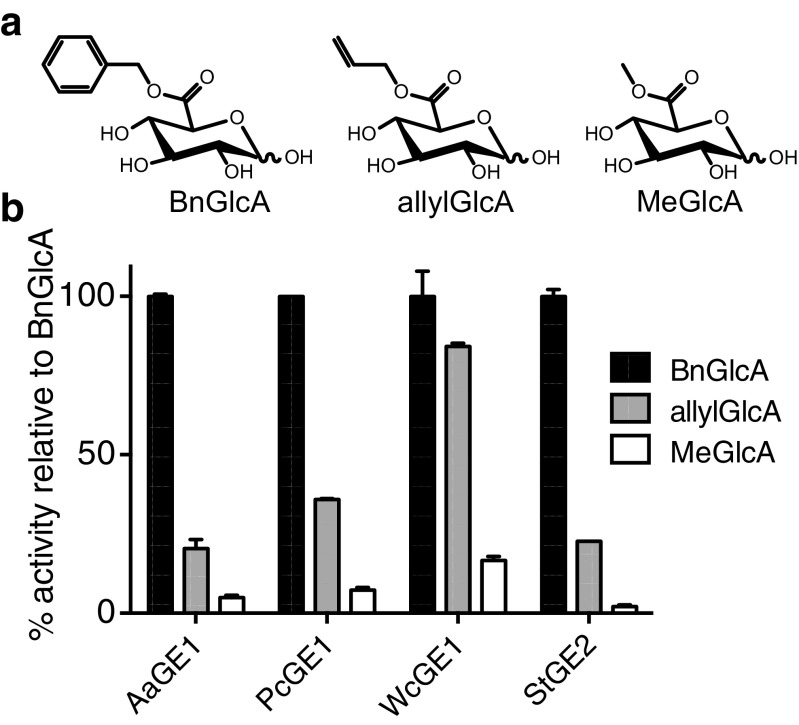
Three esters of glucuronic acid used as substrates for GEs. **a** Structures of benzyl, allyl and methyl esters of glucuronic acid (BnGlcA, allylGlcA and MeGlcA, respectively) used in this study. **b** Relative activities of AaGE1, PcGE1, WcGE1 and StGE2 on the three substrates, activity on BnGlcA was taken as 100%. *Bars* are the averages of three independent measurements; standard deviation is represented as *error bars*

To investigate the possibility of using alternative commercially available compounds for screening and activity assessment of GEs, we compared the ability of AaGE1, PcGE1 and WcGE1 to hydrolyse allyl and methyl esters of glucuronic acid (allylGlcA, MeGlcA; Fig. 7). StGE2 from *M. thermophila* and produced in *P. pastoris* was used as an additional reference. Enzyme reactions were incubated using 2 mM allylGlcA or MeGlcA as a substrate for 30 min. In all cases, enzyme action on BnGlcA produced the highest amount of hydrolysed glucuronic acid compared to the other two substrates (Fig. 7). Using WcGE1, the activity on allylGlcA reached 84% of the activity on BnGlcA. None of the other enzymes had more than 36% of the BnGlcA activity on allylGlcA. Hydrolysis of MeGlcA was even weaker; among the four tested enzymes, WcGE1 again had the highest value for this substrate (23% of BnGlcA activity).

## Discussion

Most studies of GEs to date have been conducted using synthetic substrates obtained through organic synthesis (Biely et al. 2015; D’Errico et al. 2015; Ďuranová et al. 2009a; Katsimpouras et al. 2014; Li et al. 2007; Špániková et al. 2007). The production of these compounds is laborious and requires expertise in organic chemistry, and the compounds often do not allow for high-throughput screening due to their requirement for HPLC or NMR analyses. Thus, we focused on the application of commercially available substrates that can be used to screen and characterise putative GEs. We showed the ability of three GEs—AaGE1, PcGE1 and WcGE1—to hydrolyse esters of glucuronic acid, BnGlcA, allylGlcA and MeGlcA and concomitantly demonstrated the suitability of these ester compounds as GE substrates in a spectrophotometric assay. Qualitative TLC assays with BnGlcA, allylGlcA and MeGlcA were recently reported in the characterisation of a bacterial CE15 family member (De Santi et al. 2016). In the present study, the compounds were instead used for the quantification of GE activity spectrophotometrically in microtitre plates, thereby allowing for quick and parallel measurements. Sunner et al. deemed an HPLC-based assay more suitable than the spectrophotometric assay to determine kinetic parameters of GEs on BnGlcA, since the reaction system is simpler and does not use a coupled reaction (Sunner et al. 2015). However, we demonstrated that it was possible to determine the initial enzymatic hydrolysis rates with the spectrophotometric assay for BnGlcA concentrations of up to 7 mM, which corresponds to two to four times the *K*_*m*_ value of the enzymes tested in the present work. At higher concentrations, deviations from the Michaelis-Menten kinetics were observed, likely caused by the limited solubility of the hydrophobic BnGlcA in aqueous solutions. Instability of BnGlcA at higher temperatures and alkaline pH is another limitation of using this substrate for characterisation. Nevertheless, BnGlcA proved to be a useful GE substrate if the assay conditions are chosen to accommodate for substrate instability (Sunner 2016), for example running assays at pH 6.0 and maximum 35 °C to minimise autohydrolysis. This instability issue is not exclusive to BnGlcA and has also been reported for lignin-carbohydrate complex (LCC) model compounds (Nylander et al. 2015; D’Errico et al. 2016).

We identified and produced six novel CE15 domain containing proteins, of which AaGE1 and WcGE1 were active on BnGlcA, allylGlcA and MeGlcA. In addition, we also produced and tested PcGE1 from *P. chrysosporium*, which has been previously shown to hydrolyse the GE model substrates methyl 4-O-methyl-d-glucopyranuronate and 4-nitrophenyl-2-O-(methyl-4-O-methyl-α-d-glucopyranosyluronate)-β-d-xylopyranoside (Ďuranová et al. 2009b). Four of the *Pichia* culture filtrates containing CE15 candidates did not show activity on either BnGlcA (Fig. 3), allylGlcA or MeGlcA. The genes encoding them could be inactive pseudogenes. However, multiple sequence alignment with known GEs showed that the conserved CE15 family motif, containing the catalytic serine G-C-S-R-x-G (Topakas et al. 2010), as well as the catalytic histidine are present (Fig. S4). Another possibility is that the enzymes are unable to act on the tested model substrates. Recently, a CE15 family member from an arctic marine bacterium was shown to have only low activity on typical GE substrates (De Santi et al. 2016). It is likely that substrate promiscuity exists for CE15 domain containing proteins, especially for members of this family with larger evolutionary distances to already published ones. A third possibility could be that expressed proteins are not functional because they are misfolded and subject to protein degradation, a common mechanism in eukaryotic cells (Wolff et al. 2014). For the present study, we focussed on the three enzymes that were active on the model substrate BnGlcA. Further studies investigating the activities of LfGE1, LfGE2, LfGE3 and ScGE2 are required to elucidate their respective roles in nature.

All three BnGlcA-active enzymes originate from mesophilic organisms with growth optima in the range of 20 to 45 °C. Accordingly, enzyme activities of AaGE1, PcGE1 and WcGE1 declined following incubation above 40 °C (Fig. 5d). However, AaGE1 and PcGE1 exhibited only a 22 and 28% reduction in activity respectively after incubation for 6 h at 50 °C. Thus, they showed better stability than GEs from other mesophilic organisms, such as NcGE from *Neurospora crassa* (Huynh and Arioka 2016) or Cip2 from *Hypocrea jecorina* (Li et al. 2007).

In addition, AaGE1 exhibited exceptional pH stability and was most stable at neutral and alkaline pH values, showing no loss of activity when incubated at pH 11 at 4 °C for a week (Fig. 5a). To our knowledge, AaGE1 is the first GE from an alkalophilic organism.

Buffer composition had a surprisingly big influence on enzyme stability. Buffer components can affect enzymatic activity, and interaction of buffer molecules with the enzyme could result in a modulation of stability (Mahler 1961; Wong et al. 2013). However, further investigation is required to determine the effect of the buffers on the enzymes in this case.

From our data, we also concluded that AaGE1 had the highest affinity to BnGlcA among the tested enzymes, and WcGE1 had the fastest turnover of substrate to product (*k*_*cat*_) (Fig. 6, Table 2). The measured *k*_*cat*_ values are similar to those reported for glucuronic acid esters without 4-O-methylation (Špániková et al. 2007), but are one to two orders of magnitude lower than in studies with substrates containing a 4-O-methyl group on the glucuronic acid (Ďuranová et al. 2009a; Špániková et al. 2007; Vafiadi et al. 2009). This is in accordance with the suggestion that 4-O-methylation of uronic acids plays an important role in GE-substrate interaction (Špániková et al. 2007).

A comparison of different glucuronic acid esters showed a clear preference for BnGlcA over allylGlcA and MeGlcA for AaGE1, PcGE1, WcGE1 and *M. thermophila* StGE2 (Fig. 7b). This observation supports the conclusion that GEs might have a preference for glucuronic acids esterified to bulkier side groups (D’Errico et al. 2015; D’Errico et al. 2016), which is enabled by the fact that the active site of GEs is located at the surface and thus accessible to larger substrates (Pokkuluri et al. 2011). Nevertheless, the activity of WcGE1 towards allylGlcA reached 84% of its activity on BnGlcA, which was by far the highest among the tested enzymes. Future work could investigate the structural reason for WcGE1’s comparable substrate promiscuity.

In conclusion, this study shows the applicability of the commercially available model compounds BnGlcA, MeGlcA and allylGlcA in GE activity screening and characterisation experiments. Three fungal GEs were found to be active on these substrates to different degrees. The presented enzymes showed stability over a wide pH range, making them suitable as auxiliary enzymes to improve saccharification of plant biomass.

## Electronic supplementary material

This article contains online supplementary material.

ESM 1 (PDF 1255 kb).
