# Supplementary material for: Characterisation of three fungal glucuronoyl esterases on glucuronic acid ester model compounds
Source: Appl Microbiol Biotechnol. 2017 Apr 20;101(13):5301–11. doi: 10.1007/s00253-017-8266-9 (PMC5486812; doi:10.1007/s00253-017-8266-9)
Supplement: Supplementary file 1 — (PDF 1255 kb). [file 253_2017_8266_MOESM1_ESM.pdf]

Applied Microbiology and Biotechnology

Electronic supplementary material for

Characterisation of three fungal glucuronoyl esterases on glucuronic acid ester model compounds

Silvia Hüttner[a,b]*, Sylvia Klaubauf[a,b]*, Ronald P. de Vries[c], Lisbeth Olsson[a,b#]

[a] Division of Industrial Biotechnology, Department of Biology and Biological Engineering, Chalmers University of Technology, SE-412 96 Gothenburg, Sweden
[b] Wallenberg Wood Science Center, Chalmers University of Technology, SE-412 96 Gothenburg, Sweden
[c] Fungal Physiology, CBS-KNAW Fungal Biodiversity Centre & Fungal Molecular Physiology, Utrecht University, 3584 CT Utrecht, The Netherlands

# Corresponding author: lisbeth.olsson@chalmers.se, tel: +46-(0)31-772-1000, fax: +46-31-772-3801
* These authors contributed equally to this work

Material and methods

Protein identification

For peptide sequencing, purified AaGE1 was digested with either trypsin or chymotrypsin using the filter-aided sample preparation (FASP) method (Wiśniewski et al. 2009). Samples were desalted using PepClean C18 spin columns (Thermo Fisher Scientific) according to the manufacturer's guidelines prior to analysis on Orbitrap Fusion Tribrid mass spectrometer (Thermo Fisher Scientific) interfaced with Easy nLC I000 liquid chromatography system. Peptides were separated using an in-house constructed C18 analytical column (300x0.075 mm I.D., 3 µm, Dr. Maisch, Germany) using a gradient from 5% to 25% acetonitrile in 0.1% formic acid for 30 min and finally from 25% to 80% acetonitrile in 0.1% formic acid for 5 min. Precursor ion mass spectra were acquired at 120 000 resolution and MS/MS analysis was performed in a data-dependent mode where CID spectra of the most intense precursor ions were recorded in ion trap at collision energy setting of 30 for 3 s ('top speed' setting). Charge states 2 to 7 were selected for fragmentation, dynamic exclusion was set to 30 s.
Data analysis was performed utilizing Proteome Discoverer version 1.4 (Thermo Fisher Scientific) against a database including the protein sequence. Mascot 2.3.2.0 (Matrix Science) was used as a search engine with precursor mass tolerance of 5 ppm and fragment mass tolerance of 500 mmu. Tryptic and semi-tryptic (peptide with one tryptic and one non-enzymatic cleavage site) peptides were accepted with one missed cleavage, while for chymotrypsin three missed cleavages were accepted. Methionine oxidation and cysteine alkylation were set as variable modifications. The detected peptide threshold in the software was set to 1% false discovery rate by searching against a reversed database and identified proteins were grouped by sharing the same sequences to minimise redundancy.

Table S1. Proteins used for phylogenetic analysis.

Protein	Organism	Phylum[1]	Order, family (according to NCBI TaxonomyBrowser)	Locus tag/protein ID[2]	CBM[4]	Uniprot ID	Reference
A) Previously characterised CE15 proteins (in chronological order).							
Cip2	*Trichoderma reesei*	A	Hypocreales, Hypocreaceae	AAP57749	CBM1	G0RV93	Li et al., 2007
PcGE1	*Phanerochaete chrysosporium*	B	Polyporales, Phanerochaetaceae	jgi130517 v. Phchr1, jgi3007273 v. Phchr2	CBM1	P0CT87	Ďuranová et al., 2009a,b
PcGE2				jgi6482 v. Phchr1, jgi2912243 v. Phchr2.2		P0CT88	Ďuranová et al., 2009a,b
StGE2	*Myceliophthora thermophila*	A	Sordariales, Chaetomiaceae	MYCTH_55568, jgi2308381 v. Spoth2		G2QJR6	Topakas et al., 2010
PcGCE	*Phanerochaete carnosa*	B	Polyporales, Phanerochaetaceae	AFM93784, jgi247750 v. Phaca1		K5XDZ6	Tsai et al., 2012
rScGE	*Schizophyllum commune*	B	Agaricales, Schizophyllaceae	XP_003026289, jgi238770 v. Schco1, jgi2754043 v. Schco3		D8QLP9	Wong et al., 2012
PaGE1	*Podospora anserina*	A	Sordariales, Lasiosphaeriaceae	XP_001903136, jgi2637 v. Podan2	CBM1	B2ABS0	Katsimpouras et al., 2014
CuGE	*Cerrena unicolor*	B	Polyporales, Polyporaceae	AIY68500, jgi364105 v. Cerun2	CBM1	A0A0A7EQR3	d'Errico et al., 2015
cesA	*Ruminococcus flavefaciens*	Bac	Firmicutes, Clostridiales, Ruminococcaceae	CAB55348		Q9RLB8	Biely et al., 2015
MZ0003	uncultured bacterium	Bac		CDW92038			deSanti et al., 2016
NcGE	*Neurospora crassa*	A	Sordariales, Sordariaceae	NCU09445.7		Q7S1X0	Huynh et al., 2016
B) CE15 proteins selected in this study (in alphabetical order).							
AaGE1	*Acremonium alcalophilum*	A	Glomerellales, Plectosphaerellaceae	AOT21131, jgi1078265 v. Acral2	CBM1		this study
LfGE1	*Lentithecium fluviatile*	A	Pleosporales, Lentitheciaceae	jgi384728 v. Lenfl1			this study
LfGE2				jgi311197 v. Lenfl1			this study
LfGE3				jgi349146 v. Lenfl1			this study
PcGE1	*Phanerochaete chrysosporium*	B	Polyporales, Phanerochaetaceae	jgi130517 v. Phchr1, jgi3007273 v. Phchr2	CBM1		Martinez et al., 2004, this study
ScGE2	*Schizophyllum commune*	B	Agaricales, Schizophyllaceae	jgi2517462 v. Schco3		D8QLS2	Ohm et al., 2010, this study
WcGE1	*Wolfiporia cocos*	B	Polyporales, Coriolaceae	BK009982, jgi23632 v. Wolco1			Floudas et al., 2012, this study
C) Additional CE15 proteins covering a wide taxonomic range (in alphabetical order).							
	Acremonium alcalophilum	A	Glomerellales, Plectosphaerellaceae	Acral2_1078978[3]			
	Alternaria alternata	A	Pleosporales, Pleosporaceae	XP_018385058		A0A177DJU8	
	Arthrobotrys oligospora	A	Orbiliales, Orbiliaceae	EGX47404	CBM1	G1XHL6	
	Aspergillus fumigatus	A	Eurotiales, Aspergillaceae	EDP55347, jgi99197 v. Aspfu_A1163_1		B0XMA3	Nierman et al., 2005
	Botryobasidium botryosum	B	Cantharellales, Botryobasidiaceae	KDQ18272	CBM1	A0A067N2B1	
				KDQ11090	CBM1	A0A067M8Q8	
				KDQ19074	CBM1	A0A067MTY7	
	Cerrena unicolor	B	Polyporales, Polyporaceae	jgi392659 v. Cerun2			
	Dactylellina haptotyla	A	Orbiliales, Orbiliaceae	XP_011114526	CBM1	S8A3X4	
	Glarea lozoyensis	A	Helotiales, Helotiaceae	XP_008087696, GLAREA_02289		S3CIR1	
		A		XP_008084971, GLAREA_04403		S3CM63	
	Grifola frondosa	B	Agaricales, Schizophyllaceae	OBZ79634			
	Leptosphaeria maculans	A	Pleosporales, Leptosphaeriaceae	XP_003844265, LEMA_P019160.1		E5AAU5	
				CBX90574		E4ZH04	
	Magnaporthe oryzae	A	Magnaporthales, Magnaporthaceae	MGG_03128		G4NAW0	
	Myceliophthora thermophila	A	Sordariales, Chaetomiaceae	MYCTH_96309, jgi2119719 v. Spoth2		G2QII5	Berka et al., 2011
	Neofusicoccum parvum	A	Botryosphaeriales, Botryosphaeriaceae	XP_007585921		R1EGG4	
	Oidiodendron maius	A	Leotiomycetes, Myxotrichaceae	KIN02722, jgi40592 v. Oidma1	CBM1	A0A0C3HK64	Kohler et al., 2015
				Oidma1_44280[3]			
	Parastagonospora nodorum	A	Pleosporales, Phaeosphaeriaceae	SNOG_09306, jgi2908 v. Stano2		Q0UG08	
	Penicillium chrysogenum	A	Eurotiales, Aspergillaceae	Pc13g07350, CAP91804		B6H3U7	
	Piriformospora indica / Serendipita indica	B	Sebacinales, Serendipitaceae	CCA74892, PIIN_08862	CBM1	G4TU99	
	Phanerochaete chrysosporium	B	Polyporales, Phanerochaetaceae	Phaca1_157044[3]			
				Phaca1_179285[3]			
	Podospora anserina	A	Sordariales, Lasiosphaeriaceae	PODANS_5_11620, jgi7888 v. Podan2		B2API8	Espagne et al., 2008
				Pa_1_780, jgi25 v. Podan2		A0A090CBG6	Espagne et al., 2008
	Pseudogymnoascus verrucosus	A	Leotiomycetes, Pseudeurotiaceae	XP_018132613, VE01_03452	CBM1		
	Rhytidhysteron rufulum	A	Hysteriales, Hysteriaceae	jgi111612 v. Rhyru1_1			Ohm et al., 2012
				jgi116370 v. Rhyru1_1			
	Sugiyamaella lignohabitans	A	Saccharomycetales, Trichomonascaceae	ANB15160, AWJ20_2784		A0A161HH17	

[1] A: Ascomycota, B: Basidiomycota, Bac: Bacteria

[2] Protein identifiers are given: NCBI accession numbers or locus tags, or U.S. Department of Energy Joint Genome Institute (DOE JGI) identifiers including genome version

[3] These proteins were not included in the final phylogenetic tree, because their gene models could not be verified

[4] CBM: carbohydrate-binding module

AaGE1 (full length)
Length: 496 aa
Molecular weight (incl. signal peptide, excl. N-glycans): 51.6 kDa
Molecular weight (excl. signal peptide, excl. N-glycans): 49.8 kDa

```
> AaGE1
1         MKSTVASALLVLAGTAVQAQSGPWQQCGGIGWQGPFTCVSGHTCQVLNDWYHQCVPGGGPSPPPTSPPPTTPPPT
76        SPPPTSPPPTSPPPTSPPPTSPPPTSPPPTSPPPTSPPPTSPPPTSPPPSSGSCPSTPGGLGSGNQRLPDPFTFH
151       NGNTVTSAADFQCRQREVSSLIQQYELGQFPAPPQSVTSSYSGNTLSITVSDQGRSISFSVSISGGSGSKSPAII
226       AYGAPSIPVPNGVATIRFNNDDIAAQQSGSSRGQGKFYNLYGSGHSAGAMTAWAWGVARIIDALEKTPAAGIDPT
301       RVGVTGCSRNGKGAMVAGALEPRIALTIPQESGSGGSACWRISNWQGQQGQNVQTPAQIITENVWLGPVFNNHAN
376       NVNALPFDHHQLAGLIAPRALYVIENSDMEWLGWTATYGCMAAARTQWEALGALDNFGFSQVGGNQHCSFNSGKQ
451       SAELNAFINKFLLQSGGGTTSILRTERNHGSFNLAEWTPWNVPNLR*
```

LfGE1 (full length)
Length: 404 aa
Molecular weight (incl. signal peptide, excl. N-glycans): 43.1 kDa
Molecular weight (excl. signal peptide, excl. N-glycans): 41.3 kDa

```
> LfGE1
1         MRSDILTFAALLVAAHGAPTPESVFERMLDLLPRQANVTCPATPNPMPKAAACPSVKTMPDPFLYLDGKTRVQSK
76        EEWYQCRQPEIMKLLQEYQYGYYPDHSKETVTATRSGTKLDVSIATGGKTATISATVNLPSGTGPFPVVIAIGGI
151       DNNSYLKAGIAVVTFDYGKVAADSNSKTGSFWTLYNGKDIGVLTAWAWGFHRVLDGIELKVPEIDAKKSGVTGCS
226       RLGKAALAAGLFDARIAVTMPMCSGVQGAGPYRYSLSGQGENLENSKSGAGWWTSSGISQFVGKSTQLPYDAHTI
301       VAAIAPRAVILEQGASDQFTDSKGTATVTFPAAKAVYNWLGVGPQLGMAIPKGGHCDMSGYADVLPFVQKILQGK
376       TTTRNYDDLGSWKAMPEAYPWATDLPKGK*
```

LfGE2 (full length)
Length: 388 aa
Molecular weight (incl. signal peptide, excl. N-glycans): 40.5 kDa
Molecular weight (excl. signal peptide, excl. N-glycans): 38.7 kDa

```
> LfGE2
1         MKYSIILPFLATTVLAAPLESRQAACNVPTTFPTTANSKLPNPFKFFNGGDVKTKADFECKNKEVSAAIQAQELG
76        VFPPKPSTVTASLSGSTLSISSTEGGKTVSFTVSIKTPSGNGPFPAIIAYGAASIPVPAGVATITFNNDNIGAQQ
151       GQSSRGQGKFFDLYGSGHSAGALTAWAWGVDRVMDALEATSATKIDPKRVGVTGCSRNGKGAFVAAALVGRIALG
226       LPQESGSGGAACWRVSDSEKAKGKNIQTASQIVGENVWFSKNFNAFSTKANTLATDHHQLAGMVAPRGLLVIENE
301       IDWLGPVSTTACMKAGRLIYKALGVPDNMGFTGSGNHAHCQFPSNQQSDLTAYINKFLLNQDANTANIEKGPSAD
376       VASYIDWTAPTLT*
```

LfGE3 (full length)
Length: 387 aa
Molecular weight (incl. signal peptide, excl. N-glycans): 42.2 kDa
Molecular weight (excl. signal peptide, excl. N-glycans): 40.5 kDa

```
> LfGE3
1         MLALLLPLLFSVPSLGQAPSCPNLPASINYAANPKLPDPFLALSGTRLSKKDQWPCRKEEIRQLFQRYSYGTFPP
76        RPESVTAAMSGNALKITVSEGSKSMSFSVNIKLPSSGAAPYPAIIAYGSASLPIPNTVATITYQNFEMAADNGRG
151       KGKFYEFYGSNHNAGGMIAAAWGVDRIIDALEMTPAAKIDPKRVGVTGCSRNGKGSMIAGAFVDRIALALPQEGG
226       QSAAGCWRIADEIQKNGTKVETAHQIVNGDSWFSTDFSKYVDTVPTLPWDNHMLHALYAYPPRGLLIIENTAIDY
301       LGPTSNYHCATAGRKVHEALGVKDYFGFSQNSHSDHCGFPKAQQPELTAFIERFLLAKDTKTDVWKTDGKFTIDE
376       RRWIDWAVPSLS*
```

PcGE1 (full length)
Length: 472 aa
Molecular weight (incl. signal peptide, excl. N-glycans): 49.2 kDa
Molecular weight (excl. signal peptide, excl. N-glycans): 47.2 kDa

```
> PcGE1
1         MKSAAYLAALAAVLPAYVNAQAQEWGQCGGIGWTGATTCVSGTVCTVLNPYYSQCLPGTATTAPPPPPPPPTSVS
76        SSSSSSTSSAPPSGPSGTSPTCSVASTIPGFSNAALPNPFVFNDGSPVQSKADFTCRQQQILALIQGYEAGALPG
151       PPQSVTASFSKSGSTGTLSITVTDNGKSISFAPTISIPSGTPPANGWPLVIAFEGGSIPIPAGIAKLTYSNSDMA
226       QQTDTSSRGKGLFYNLYGSGATASAMTAWAWGVSRIIDALEKTPSAQINTQRIAVTGCSRDGKGALMAGALEPRI
301       ALTIPQESGSGGDTCWRLSKAESDQGHQVQTATEIVTENVWFSTNFNNYVNNLNVLPYDHHMLMALVAPRALVSF
376       ENTDYTWLSPMSAWGCVNAAHTVFSALGVADHHGFAQVGGHAHCAWPDSLTPSLNAFFNRFLLDQNVDTNVFTTN
451       NQFGGATWTQSSWINWSTPTLS*
```

ScGE2 (full length)
Length: 389 aa
Molecular weight (incl. signal peptide, excl. N-glycans): 41.6 kDa
Molecular weight (excl. signal peptide, excl. N-glycans): 39.5 kDa

```
> ScGE2
1         MLLASLVLTFFFFERARAQCAELPSTLPGVDDLDALNTLPDPFTFYNGTPLSSVDDWACRQQEIKQLVQHYFYGY
76        LPDTSDEANPPLQTVTAKLSSGWQGQTLAITIASGSKSASFNATLTLPSGASADAPVPAIITLGGFGPSATGAAA
151       VSFDAASVAYDGPLKTGAFWDLYEGDIGVLAAWGWGFSKIIDALEAAVPEVDIERIAVSGCSRYGKAALAAGFFD
226       ERVTLTVPMSSGLMGMAPFRFQYEENGANEQLSDMDDTSSSWPDDTLVSFRNDPARLPVDSNFITAGVAPRALIW
301       DEGTTDYWTNPEGTAAVTFPATLALYEWLGAGDNVGIALRNSGHCDPSGNTNVADFINKVFFGTETDRDYHDISP
376       FTAHEETFPFAAPE*
```

WcGE1 (full length)
Length: 408 aa
Molecular weight (incl. signal peptide, excl. N-glycans): 44.1 kDa
Molecular weight (excl. signal peptide, excl. N-glycans): 42.2 kDa

```
> WcGE1
1         MASSSRFAALLLLALPALALPPSQVVPRAACATPSTVPGYNNDRLPDPFLFDDGTAVTSSADWDCRRSQIAAVVQ
76        GYEAGYLPPQPPIVSATFSSSDGTGTLTVTAGLSSDNTISFSEPITYPSGTAPAAGWPLVIAYDVLSIPVPDGIA
151       VMVYNNDDIAQENDLSSRGVGLFYDLYGTDATASAMTAWVWGVSRIIDALETTPAANINTAKIAVTGCSRDGKGA
226       LMAGAFEPRVALTIPQESGSGGDTCWRLSKYEQDSGDVVQQATEIVTENVWFSTNFDNYVNNLSVLPYDHHELAA
301       MVAPRPLLSYENTEYEWLSPLSAYGCMSAAHTVYEALGIPDYHGFVQVGNHSHCYFPDTLDDSLYAFFDRFLLDE
376       DDVSTDYFTTNYQFNGTVWNASYWINWTTPQLD*
```

Signal peptide (predicted by SignalP v4.1)
CBM1 (predicted by dbCAN v4.0)
Proline-rich linker region
CE15 domain (predicted by dbCAN v4.0)
N-glycosylation site (predicted by NetNGlyc 1.0)

Figure S1. Protein sequences and annotation of putative fungal GEs

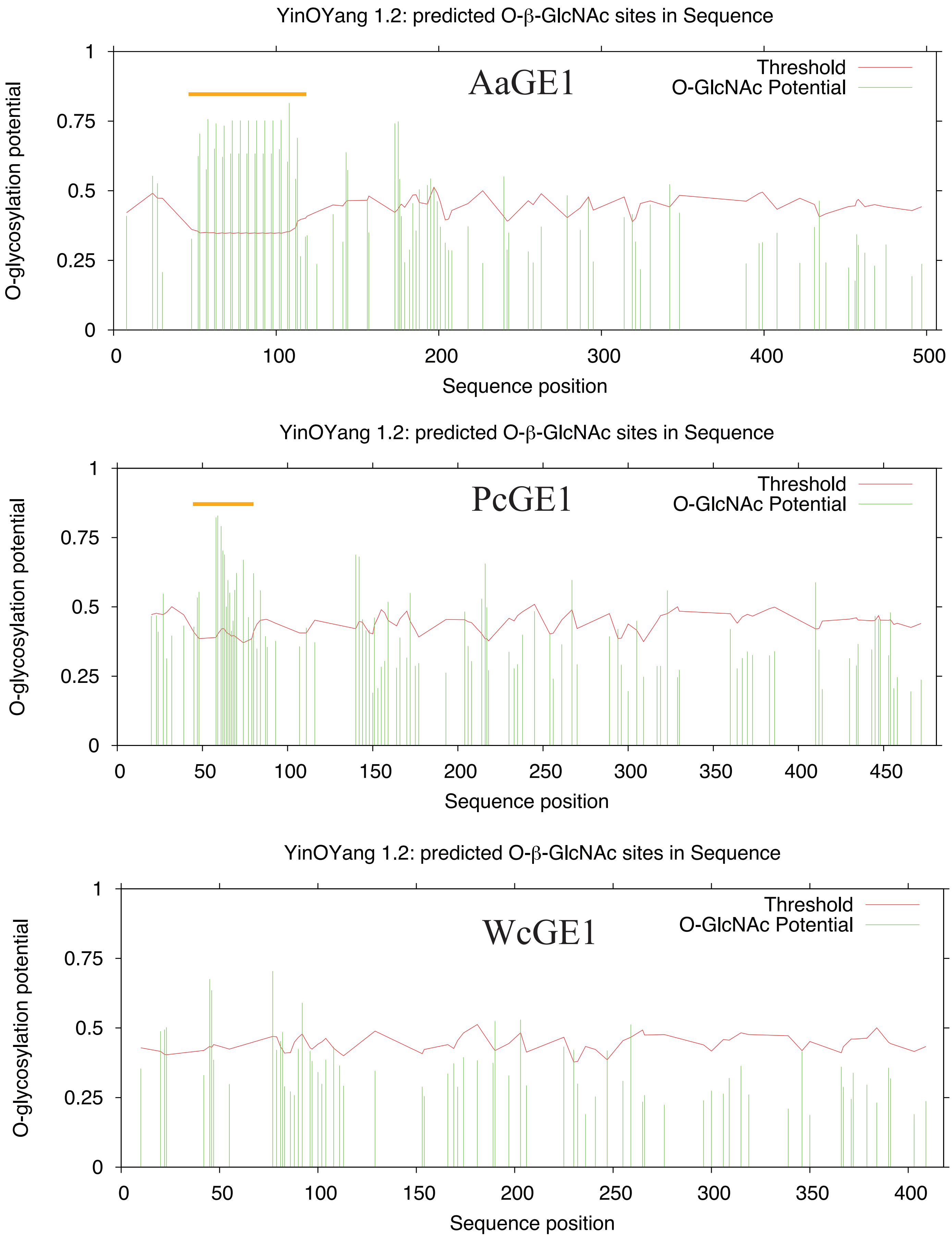

Figure S2. Prediction of O-glycosylation of GEs with YinOYang 1.2. The orange bar indicates the location of the proline-rich linker region between CBM1 and catalytic CE15 domain.

```
 EAEAEFQSGPWQQCGGIGWQGPFTCVSGHTCQVLNDWYHQCVPGGGPSPPPTSPPPTTPPPTSPPPTSPPPTSPPPTSPPPTSPPPTSPPPTSPPPTSPP
1                                                                                                   100
 PTSPPPTSPPPSSGSCPSTPGGLGSGNQRLPDPFTFHNGNTVTSAADFQCRQREVSSLIQQYELGQFPAPPQSVTSSYSGNTLSITVSDQGRSISFSVSI
101                                                                                                 200
 SGGSGSKSPAIIAYGAPSIPVPNGVATIRFNNDDIAAQQSGSSRGQGKFYNLYGSGHSAGAMTAWAWGVARIIDALEKTPAAGIDPTRVGVTGCSRNGKG
201                                                                                                 300
 AMVAGALEPRIALTIPQESGSGGSACWRISNWQGQQGQNVQTPAQIITENVWLGPVFNNHANNVNALPFDHHQLAGLIAPRALYVIENSDMEWLGWTATY
301                                                                                                 400
 GCMAAARTQWEALGALDNFGFSQVGGNQHCSFNSGKQSAELNAFINKFLLQSGGGTTSILRTERNHGSFNLAEWTPWNVPNLRGLEQKLISEEDLNSAVD
401                                                                                                 500
 HHHHHH
501    506
```

Figure S3. Protein sequencing of AaGE1. The complete sequence of secreted AaGE1 (i.e. without signal peptide) is shown. Peptides (created by digestion with chymotrypsin and trypsin) found and matched to the AaGE1 sequence are underlined. The proline-rich linker region between CBM1 and the catalytic CE15 domain is shaded in grey. Percentage of covered parts: 73%. Of the 139 aa not covered, one stretch of 90 aa stretches over most of the proline-rich linker region.

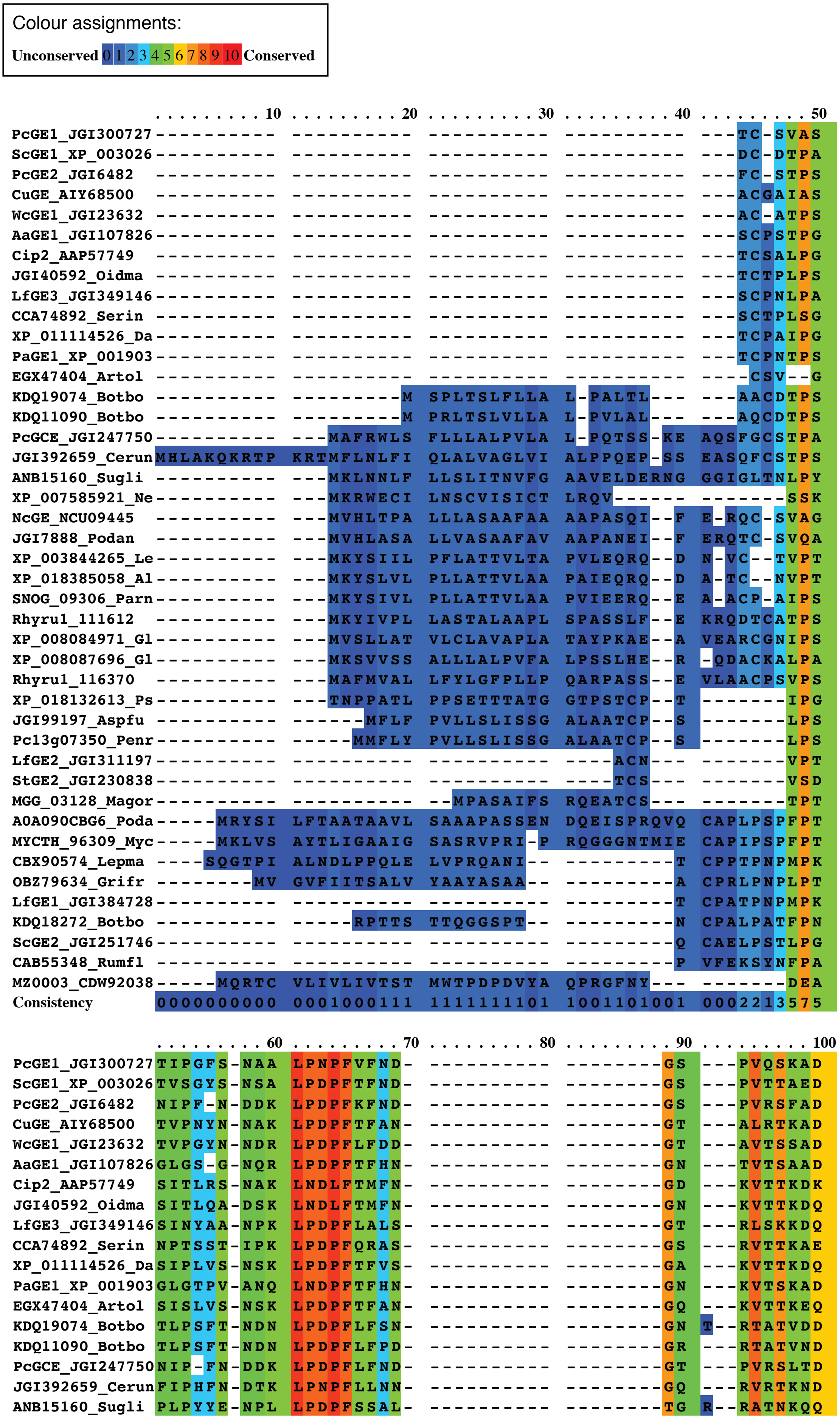

Colour assignments:
Unconserved 0 1 2 3 4 5 6 7 8 9 10 Conserved
. . . . 10 . . . . 20 . . . . 30 . . . . 40 50
PcGE1_JGI300727 ---------- ---------- ---------- ---------- ---TC-SVAS
ScGE1_XP_003026 ---------- ---------- ---------- ---------- ---DC-DTPA
PcGE2_JGI6482 ---------- ---------- ---------- ---------- ---FC-STPS
CuGE_AIY68500 ---------- ---------- ---------- ---------- ---ACGAIAS
WcGE1_JGI23632 ---------- ---------- ---------- ---------- ---AC-ATPS
AaGE1_JGI107826 ---------- ---------- ---------- ---------- ---SCPSTPG
Cip2_AAP57749 ---------- ---------- ---------- ---------- ---TCSALPG
JGI40592_Oidma ---------- ---------- ---------- ---------- ---TCTPLPS
LfGE3_JGI349146 ---------- ---------- ---------- ---------- ---SCPNLPA
CCA74892_Serin ---------- ---------- ---------- ---------- ---SCTPLSG
XP_011114526_Da ---------- ---------- ---------- ---------- ---TCPAIPG
PaGE1_XP_001903 ---------- ---------- ---------- ---------- ---TCPNTPS
EGX47404_Artol ---------- ---------- ---------- ---------- ----CSV--G
KDQ19074_Botbo ---------- ---------M SPLTSLFLLA L-PALTL--- ---AACDTPS
KDQ11090_Botbo ---------- ---------M PRLTSLVLLA L-PVLAL--- ---AQCDTPS
PcGCE_JGI247750 ---------- ---MAFRWLS FLLLALPVLA L-PQTSS-KE AQSFGCSTPA
JGI392659_Cerun MHLAKQKRTP KRTMFLNLFI QLALVAGLVI ALPPQEP-SS EASQFCSTPS
ANB15160_Sugli ---------- ---MKLNNLF LLSLITNVFG AAVELDERNG GGIGLTNLPY
XP_007585921_Ne ---------- ---MKRWECI LNSCVISICT LRQV------ -------SSK
NcGE_NCU09445 ---------- ---MVHLTPA LLLASAAFAA AAPASQI--F E-RQC-SVAG
JGI7888_Podan ---------- ---MVHLASA LLVASAAFAV AAPANEI--F ERQTC-SVQA
XP_003844265_Le ---------- ---MKYSIIL PFLATTVLTA PVLEQRQ--D N-VC--TVPT
XP_018385058_Al ---------- ---MKYSLVL PLLATTVLAA PAIEQRQ--D A-TC--NVPT
SNOG_09306_Parn ---------- ---MKYSIVL PLLATTVLAA PVIEERQ--E A-ACP-AIPS
Rhyru1_111612 ---------- ---MKYIVPL LASTALAAPL SPASSLF--E KRQDTCATPS
XP_008084971_Gl ---------- ---MVSLLAT VLCLAVAPLA TAYPKAE--A VEARCGNIPS
XP_008087696_Gl ---------- ---MKSVVSS ALLLALPVFA LPSSLHE--R -QDACKALPA
Rhyru1_116370 ---------- ---MAFMVAL LFYLGFPLLP QARPASS--E VLAACPSVPS
XP_018132613_Ps ---------- ---TNPPATL PPSETTTATG GTPSTCP--T -------IPG
JGI99197_Aspfu ---------- ------MFLF PVLLSLISSG ALAATCP--S -------LPS
Pc13g07350_Penr ---------- -----MMFLY PVLLSLISSG ALAATCP--S -------LPS
LfGE2_JGI311197 ---------- ---------- ---------- ----ACN--- -------VPT
StGE2_JGI230838 ---------- ---------- ---------- ----TCS--- -------VSD
MGG_03128_Magor ---------- ---------- --MPASAIFS RQEATCS--- -------TPT
A0A090CBG6_Poda -----MRYSI LFTAATAAVL SAAAPASSEN DQEISPRQVQ CAPLPSPFPT
MYCTH_96309_Myc -----MKLVS AYTLIGAAIG SASRVPRI-P RQGGGNTMIE CAPIPSPFPT
CBX90574_Lepma ----SQGTPI ALNDLPPQLE LVPRQANI-- ---------T CPPTPNPMPK
OBZ79634_Grifr --------MV GVFIITSALV YAAYASAA-- ---------A CPRLPNPLPT
LfGE1_JGI384728 ---------- ---------- ---------- ---------T CPATPNPMPK
KDQ18272_Botbo ---------- -----RPTTS TTQGGSPT-- ---------N CPALPATFPN
ScGE2_JGI251746 ---------- ---------- ---------- ---------Q CAELPSTLPG
CAB55348_Rumfl ---------- ---------- ---------- ---------P VFEKSYNFPA
MZ0003_CDW92038 -----MQRTC VLIVLIVTST MWTPDPDVYA QPRGFNY--- -------DEA
Consistency 0000000000 0001000111 1111111101 1001101001 0002213575
. . . . 60 . . . . 70 . . . . 80 . . . . 90 100
PcGE1_JGI300727 TIPGFS-NAA LPNPFVFND- ---------- --------GS ---PVQSKAD
ScGE1_XP_003026 TVSGYS-NSA LPDPFTFND- ---------- --------GS ---PVTTAED
PcGE2_JGI6482 NIPF-N-DDK LPDPFKFND- ---------- --------GS ---PVRSFAD
CuGE_AIY68500 TVPNYN-NAK LPDPFTFAN- ---------- --------GT ---ALRTKAD
WcGE1_JGI23632 TVPGYN-NDR LPDPFLFDD- ---------- --------GT ---AVTSSAD
AaGE1_JGI107826 GLGS-G-NQR LPDPFTFHN- ---------- --------GN ---TVTSAAD
Cip2_AAP57749 SITLRS-NAK LNDLFTMFN- ---------- --------GD ---KVTTKDK
JGI40592_Oidma SITLQA-DSK LNDLFTMFN- ---------- --------GN ---KVTTKDQ
LfGE3_JGI349146 SINYAA-NPK LPDPFLALS- ---------- --------GT ---RLSKKDQ
CCA74892_Serin NPTSST-IPK LPDPFQRAS- ---------- --------GS ---RVTTKAE
XP_011114526_Da SIPLVS-NSK LPDPFTFVS- ---------- --------GA ---KVTTKDQ
PaGE1_XP_001903 GLGTPV-ANQ LNDPFTFHN- ---------- --------GN ---KVTSKAD
EGX47404_Artol SISLVS-NSK LPDPFTFAN- ---------- --------GQ ---KVTTKEQ
KDQ19074_Botbo TLPSFT-NDN LPDPFLFSN- ---------- --------GN T--RTATVDD
KDQ11090_Botbo TLPSFT-NDN LPDPFLFPD- ---------- --------GR ---RTATVND
PcGCE_JGI247750 NIP-FN-DDK LPDPFLFND- ---------- --------GT ---PVRSLTD
JGI392659_Cerun FIPHFN-DTK LPNPFLLNN- ---------- --------GQ ---RVRTKND
ANB15160_Sugli PLPYYE-NPL LPDPFSSAL- ---------- --------TG R--RATNKQQ

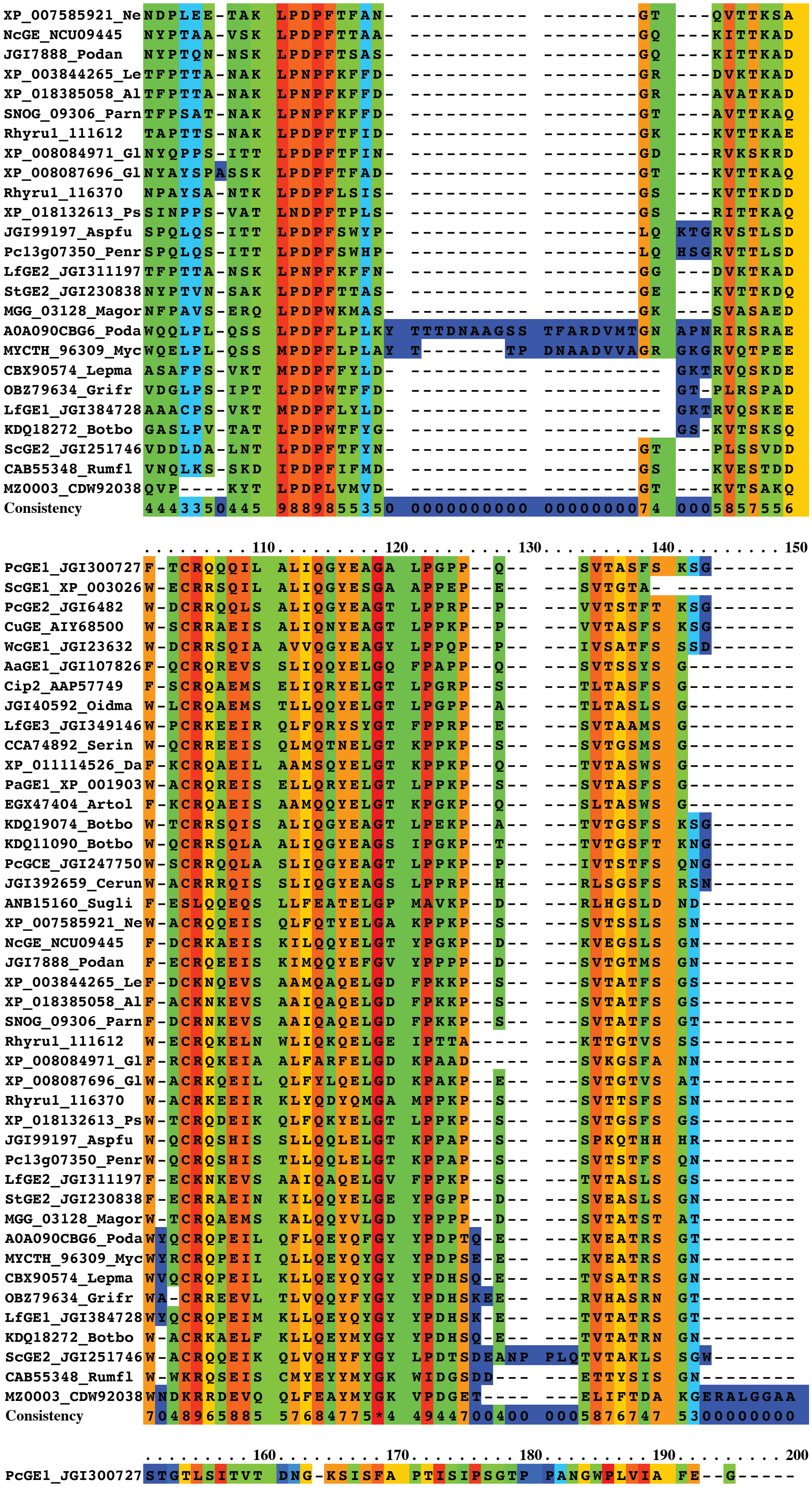

```
ScGE1_XP_003026 SGNSLSVQVS YGG-KSITFN NSITYPSGTA PAEGWPVIIA YE--F-----
PcGE2_JGI6482   TTGNLTVTAG FPG-KTITFS SPITFPTGTA PFGGWPLVIA YG--G-----
CuGE_AIY68500   NTGTLAITAG LSNSQTIKFS PTISYPSGTP PANGWPLIIA YE--G-----
WcGE1_JGI23632  GTGTLTVTAG LSSDNTISFS EPITYPSGTA PAAGWPLVIA YD--V-----
AaGE1_JGI107826 --NTLSITVS DQG-RSISFS VSISGGSG-- --SKSPAIIA YG--A-----
Cip2_AAP57749   --NTLTINCG EAG-KSISFT VTITYPSSGT --APYPAIIG YG--G-----
JGI40592_Oidma  --STLTINCG DSG-KSMSFT AAITYPSSGT --APYPAIIA FD--G-----
LfGE3_JGI349146 --NALKITVS EGS-KSMSFS VNIKLPSSGA --APYPAIIA YG--S-----
CCA74892_Serin  --NTLTINVS EAG-KSISFS ISISYPSSGT --APYPAIIA YG--A-----
XP_011114526_Da --NKLTINVS NGG-TSISFA VTITAPSGGR --SPYPAIIG YG--G-----
PaGE1_XP_001903 --STLSISVS EGG-KSISFT VSINNRPSGA --GPHPAIIN FGTFG-----
EGX47404_Artol  --NTLTINVS DQG-KSISFS VTVTAPSGGR --APYPAIIG YD--G-----
KDQ19074_Botbo  NTGTLTVTAG -DNGKSVNWA NSISYPTGTA PAGGWPLLIA YG--G-----
KDQ11090_Botbo  NSGSLTVTAS -QNGKSVNWA NSISYPSGTP PAGGWPLLIA LG--G-----
PcGCE_JGI247750 LTGNLTVTAG -FPGNTTTFS SPVTFPNGTV PTEGWPLLIA YS--G-----
JGI392659_Cerun TTANLTITAG LSADNEITFT PTISYPSGNA PKGGWPLIIG YG--G-----
ANB15160_Sugli  ---TLIITAE -VNGRSITYN ATIVYPSAG- -RAPYPAMIA YS--G-----
XP_007585921_Ne ---TLTITAS -EGGKSISFS VSISYPSSG- -TAPYPAIIA YG--Y-----
NcGE_NCU09445   ---TLTVRIT -VGSQTVSFS ASIKKPSSG- -SGPFPAIIG IG--G-----
JGI7888_Podan   ---NIQVRVT -VGSKSITFS AGIRKP-SG- -SGPFPAIIG VG--G-----
XP_003844265_Le ---SLSITST -EGGKSVSFS VSIKKP-SG- -AGPFPAIIA YG--A-----
XP_018385058_Al ---SLSITST -EGGKSVTFS VSIKKP-SG- -TGPFPAIIA YG--A-----
SNOG_09306_Parn ---SLAITST -EGGKSVSFS VTIKKP-SG- -AGPFPAIIA YG--A-----
Rhyru1_111612   ---SISVSVS -DGGKSISFT ASVRAPSGG- -SAPYPAVIA FG--G-----
XP_008084971_Gl ---ILTVTVT -NNKKSIAFN ATITYPEKG- -KAPYPAIIT LG--Y-----
XP_008087696_Gl ---SISVKVT -NAGKSISFS ATVKMPSGV- -TGAAPAIIA YG--G-----
Rhyru1_116370   ---SLKITVE -DNGKSTSFS VSIKYPSSG- -SGPFPAIIG YG--G-----
XP_018132613_Ps ---TLTINVS -EGGKSISFT ASITYPSSG- -TAPYPAIIA IG--G-----
JGI99197_Aspfu  ---HQCR--- ----KHHLLH SNHHLPLLR- -RRPYPAMIA YG--G-----
Pc13g07350_Penr ---KLSITAS -NAGKTISFT ATITYPSSG- -AGPYPAMIA YG--G-----
LfGE2_JGI311197 ---TLSISST -EGGKTVSFT VSIKTPS--G -NGPFPAIIA YG--A-----
StGE2_JGI230838 ---SITVRVT -VGSKSISFS ASIRKPS--G -AGPFPAIIG IG--G-----
MGG_03128_Magor ---GLNIQIK -VGSNTRAIS VGITKPTNPG -TSGGPAIIG IG--G-----
A0A090CBG6_Poda ---TLQITVT GPQNKRGSFR ATIQLPSGAS SSRPAPVVIN IGGMQ-----
MYCTH_96309_Myc ---TLNIVVT A-GGKQGSFR ATISLPSGAS ASNPAPVVIN IGGMQ-----
CBX90574_Lepma  ---TLTITIA A-GGKTASIA ATLTLPSGS- --GPFPVIIS IGGMD-----
OBZ79634_Grifr  ---TLNISVS V-GNRTGTFN ATLTFPNGTT RFRPVPVMIN TGGVN-----
LfGE1_JGI384728 ---KLDVSIA T-GGKTATIS ATVNLP-SG- -TGPFPVVIA IGGID-----
KDQ18272_Botbo  ---TLSISVS A-GGKTGTFS ATITLPTTG- -QAPYPVVIT PGSVD-----
ScGE2_JGI251746 QGQTLAITIA SGS-KSASFN ATLTLPSGAS ADAPVPAIIT LGGFG-----
CAB55348_Rumfl  -SMTINVKRK STG-KTASFK AVINLPKNVR HEGGAPVILG MHKGI-----
MZ0003_CDW92038 IRKQVKISFG EKE-DAPAMD LLIYLPADA- -KVRVPVFLG LNFHGNHTIH
Consistency     0016859555 1262775786 5695495541 03565*6896 5700500000

                ......... 210 ......... 220 ......... 230 ......... 240 ......... 250
PcGE1_JGI300727 ---------- ---------- ---------- --------GS IPIPAGIAKL
ScGE1_XP_003026 ---------- ---------- ---------- --------PS LPIPSNVATL
PcGE2_JGI6482   ---------- ---------- ---------- --------VS IPIPDGIAVL
CuGE_AIY68500   ---------- ---------- ---------- --------GS IPIPAGVATL
WcGE1_JGI23632  ---------- ---------- ---------- --------LS IPVPDGIAVM
AaGE1_JGI107826 ---------- ---------- ---------- --------PS IPVPNGVATI
Cip2_AAP57749   ---------- ---------- ---------- --------GS LPAPAGVAMI
JGI40592_Oidma  ---------- ---------- ---------- --------GS LPAPASVAMI
LfGE3_JGI349146 ---------- ---------- ---------- --------AS LPIPNTVATI
CCA74892_Serin  ---------- ---------- ---------- --------MS IPTPAGVAVI
XP_011114526_Da ---------- ---------- ---------- --------GS IPVPAGVALI
PaGE1_XP_001903 ---------- ---------- ---------- --------AS LPVPAGVATI
EGX47404_Artol  ---------- ---------- ---------- --------GS IPIPAGVAKI
KDQ19074_Botbo  ---------- ---------- ---------- --------GS IPVPAGIATL
KDQ11090_Botbo  ---------- ---------- ---------- --------GS IPVPAGIATL
PcGCE_JGI247750 ---------- ---------- ---------- --------LS IPIPDGIAVL
JGI392659_Cerun ---------- ---------- ---------- --------PS IPIPDGVAVM
ANB15160_Sugli  ---------- ---------- ---------- --------IS IPQPDNIAFI
XP_007585921_Ne ---------- ---------- ---------- --------PS IPIPAGVATI
NcGE_NCU09445   ---------- ---------- ---------- --------IS IPIPSTVATI
JGI7888_Podan   ---------- ---------- ---------- --------AS IPIPSNVATI
XP_003844265_Le ---------- ---------- ---------- --------AS IPVPANVATI
XP_018385058_Al ---------- ---------- ---------- --------AS LPVPSNVATI
SNOG_09306_Parn ---------- ---------- ---------- --------AS IPVPATVATI
Rhyru1_111612   ---------- ---------- ---------- --------SS LPIPAGVATI
XP_008084971_Gl ---------- ---------- ---------- --------PN IPKLDGVAFI
XP_008087696_Gl ---------- ---------- ---------- --------AS LPIPAGVATI
Rhyru1_116370   ---------- ---------- ---------- --------GS IPIPANVASI
XP_018132613_Ps ---------- ---------- ---------- --------LS IPTPAGVAVI
JGI99197_Aspfu  ---------- ---------- ---------- --------LS IPLPPGVAAI
```

```
Pc13g07350_Penr ---------- ---------- ---------- --------LS IPLPPGVATI
LfGE2_JGI311197 ---------- ---------- ---------- --------AS IPVPAGVATI
StGE2_JGI230838 ---------- ---------- ---------- --------AS IPIPSNVATI
MGG_03128_Magor ---------- ---------- ---------- --------IS IPVPAGIGRI
A0A090CBG6_Poda ---------- ---------- ---------- --------NQ PYLQAGIAVA
MYCTH_96309_Myc ---------- ---------- ---------- --------NQ PYLSAGIAVA
CBX90574_Lepma  ---------- ---------- ---------- --------VK SYTNAGIAVA
OBZ79634_Grifr  ---------- ---------- ---------- --------ET VFLGSGVALA
LfGE1_JGI384728 ---------- ---------- ---------- --------NN SYLKAGIAVV
KDQ18272_Botbo  ---------- ---------- ---------- --------NN AFLSQGVALV
ScGE2_JGI251746 ---------- ---------- ---------- ---------- -PSATGAAAV
CAB55348_Rumfl  ---------- ---------- ---------- -------SES TATSNGYAVI
MZ0003_CDW92038 KDKEIWLTES WVRTNKKFGI TKNKANELSR GVAAGRWQIE KAIAKGYGVA
Consistency     0000000000 0000000000 0000000000 0000000037 6767578957

                .........260.........270.........280.........290.........300
PcGE1_JGI300727 TYSNSDMAQQ TDTSS----- ---------- RGKGLFYNLY GSG----ATA
ScGE1_XP_003026 SFQNSAMGKQ DSTSS----- ---------- RGQGLFYDLY GSS----SNA
PcGE2_JGI6482   TYDNSAMAEQ NDQSS----- ---------- RGVGLFFDVY GAN----ATA
CuGE_AIY68500   TYSNSDMAQQ NSASS----- ---------- RGQGLFYQLY GST----HSA
WcGE1_JGI23632  VYNNDDIAQE NDLSS----- ---------- RGVGLFYDLY GTD----ATA
AaGE1_JGI107826 RFNNDDIAAQ QSGSS----- ---------- RGQGKFYNLY GSG----HSA
Cip2_AAP57749   NFNNDNIAAQ VNTGS----- ---------- RGQGKFYDLY GSS----HSA
JGI40592_Oidma  NFNCDDMAAQ VSTSS----- ---------- RGQGKFYDLY GSG----ASA
LfGE3_JGI349146 TYQNFEMAAD NG-------- ---------- RGKGKFYEFY GSN----HNA
CCA74892_Serin  TFNNDDIAAQ QNSGS----- ---------- RGSGKFYTLY GSN----HSA
XP_011114526_Da NFNNDEIAAQ TNTGS----- ---------- RGQGKFYTLY GSG----HSA
PaGE1_XP_001903 NFNNDDIAQQ QGGSS----- ---------- RGRGKFYDLY GSS----HSA
EGX47404_Artol  SFSIADIAAQ TNTGS----- ---------- RGQGKFYNLY GSG----HSA
KDQ19074_Botbo  NYNNDNIGQQ NSGSS----- ---------- RGVGLFYNLY GSS----ATA
KDQ11090_Botbo  NYNNDNIAQQ SSTSS----- ---------- RGTGLFYNLY GNT----ATA
PcGCE_JGI247750 TYDNSAIGEQ NDQTS----- ---------- RGVGQFFDVY GHN----ATA
JGI392659_Cerun FYDNSGMAQQ NDLSS----- ---------- RGVGLFYDLY GRN----ASS
ANB15160_Sugli  VFDNSNFAQQ NAMYSASGIA YGGGDAFTTT RGQGQFYDLF GAA----HPS
XP_007585921_Ne TFNNDDIAAQ TNTGS----- ---------- RGQGKFYTLY GSG----HGA
NcGE_NCU09445   TFPNDDFAQQ SGTSS----- ---------- RGRGKFYTLF GSS----HSA
JGI7888_Podan   TFGNDAFGAQ SGMGS----- ---------- RGRGQFYDLF GSS----HSA
XP_003844265_Le TFNNDEIGQQ QGGNS----- ---------- RGKGKFFDLY GSS----HSA
XP_018385058_Al TFNNDEIGQQ QGGSS----- ---------- RGKGKFFDLY GSS----HSA
SNOG_09306_Parn TFNNDDIGQQ SGGSS----- ---------- RGKGKFYDLY GSS----HSA
Rhyru1_111612   TFNNDQMGAQ SGGSS----- ---------- RGKGKFYDLY GSG----HSA
XP_008084971_Gl NFPNDQIAGQ IGASY----- ---------- RGQGLFYELY GKN----ASA
XP_008087696_Gl TFNNDGVAQQ TNSGS----- ---------- RGKGLFYDLY GSG----HSA
Rhyru1_116370   SYSNFDIGAD NG-------- ---------- RGKGKFYDIY GKD----HSA
XP_018132613_Ps NFGNDDIAAQ NSGSS----- ---------- RGQGKFYTIY GAG----HSA
JGI99197_Aspfu  TFDNSQIAQQ NDQSS----- ---------- RGQGLFYTLY GAN----HSA
Pc13g07350_Penr TFDNSQIAQQ NDQSS----- ---------- RGKGLFYTLY GAD----HPA
LfGE2_JGI311197 TFNNDNIGAQ QGQSS----- ---------- RGQGKFFDLY GSG----HSA
StGE2_JGI230838 TFNNDEFGAQ MGSGS----- ---------- RGQGKFYDLF GRD----HSA
MGG_03128_Magor NFGNDACASQ AGSQS----- ---------- HGTGWFFDLH GRS----HSA
A0A090CBG6_Poda QFDYTTVAPD SNSK------ ---------- --TGVFWSIY N-G----RDI
MYCTH_96309_Myc QFDYTTVSPD SNAK------ ---------- --TGAFWSIY N-G----RDI
CBX90574_Lepma  AFDYTKVAAD SNSK------ ---------- --SGSFWTLY N-G----KDI
OBZ79634_Grifr  TFDVDSVAAD STTP------ ---------- --GGAFWDLY S-G----EDI
LfGE1_JGI384728 TFDYGKVAAD SNSK------ ---------- --TGSFWTLY N-G----KDI
KDQ18272_Botbo  SFSVTAVAAD SASK------ ---------- --TGAFWSVY N-G----RNI
ScGE2_JGI251746 SFDAASVAYD GP-------- ---------- LKTGAFWDLY E------GDI
CAB55348_Rumfl  TYDSDGMFSA PGTAQ----- -------DNN QHKGAFYDLY PYGRNWDEQT
MZ0003_CDW92038 TIYCGDIDPD FNFPS----- ---------N GIQAYYYKKD QTIP-EKGQW
Consistency     6856547757 4544500000 0000000000 6659497688 7450000567

                                              CE15 consensus: G-C-S-R-x-G
                .........310.........320.........330.........340.........350
PcGE1_JGI300727 SAMTAWAWGV SRIIDALEKT P--SAQINTQ RIAVTGCSRD GKGALMAGAL
ScGE1_XP_003026 SAMTAWAWGV SRIIDAIEST P--DAKLNPA AVGVTGCSRN GKGALMAGAL
PcGE2_JGI6482   SSMTAWVWGL SRIIDSLEVT P--AAHINTA KIAVTGCSRN GKGALMAGAF
CuGE_AIY68500   SAMTAWVWGV SRIIDALEMT P--TAQINTQ RIGVTGCSRD GKGALMAGAF
WcGE1_JGI23632  SAMTAWVWGV SRIIDALETT P--AANINTA KIAVTGCSRD GKGALMAGAF
AaGE1_JGI107826 GAMTAWAWGV ARIIDALEKT P--AAGIDPT RVGVTGCSRN GKGAMVAGAL
Cip2_AAP57749   GAMTAWAWGV SRVIDALELV P--GARIDTT KIGVTGCSRN GKGAMVAGAF
JGI40592_Oidma  GAMTAWAWGV SRVIDALEIT S--SAKIDTT RIGVTGCSRN GKGAMVAGAF
LfGE3_JGI349146 GGMIAAAWGV DRIIDALEMT P--AAKIDPK RVGVTGCSRN GKGSMIAGAF
CCA74892_Serin  GALIAWAWGV SRIIDAIETT T--NVRINPA KIGVTGCSRN GKGAIVAGAF
XP_011114526_Da GALTAWAWGV SRIIDALEIT P--AAGVNPA RVGVTGCSRN GKGAIVAGAL
PaGE1_XP_001903 GALTAWAWGV SRIVDALELT Q-AQTGIDPT RLGVTGCSRN GKGAIVAGAL
EGX47404_Artol  GALTAWAWGV SRIIDAIEIT P--ATNINP  KIGVTGCSRN GKGAFVAGAL
```

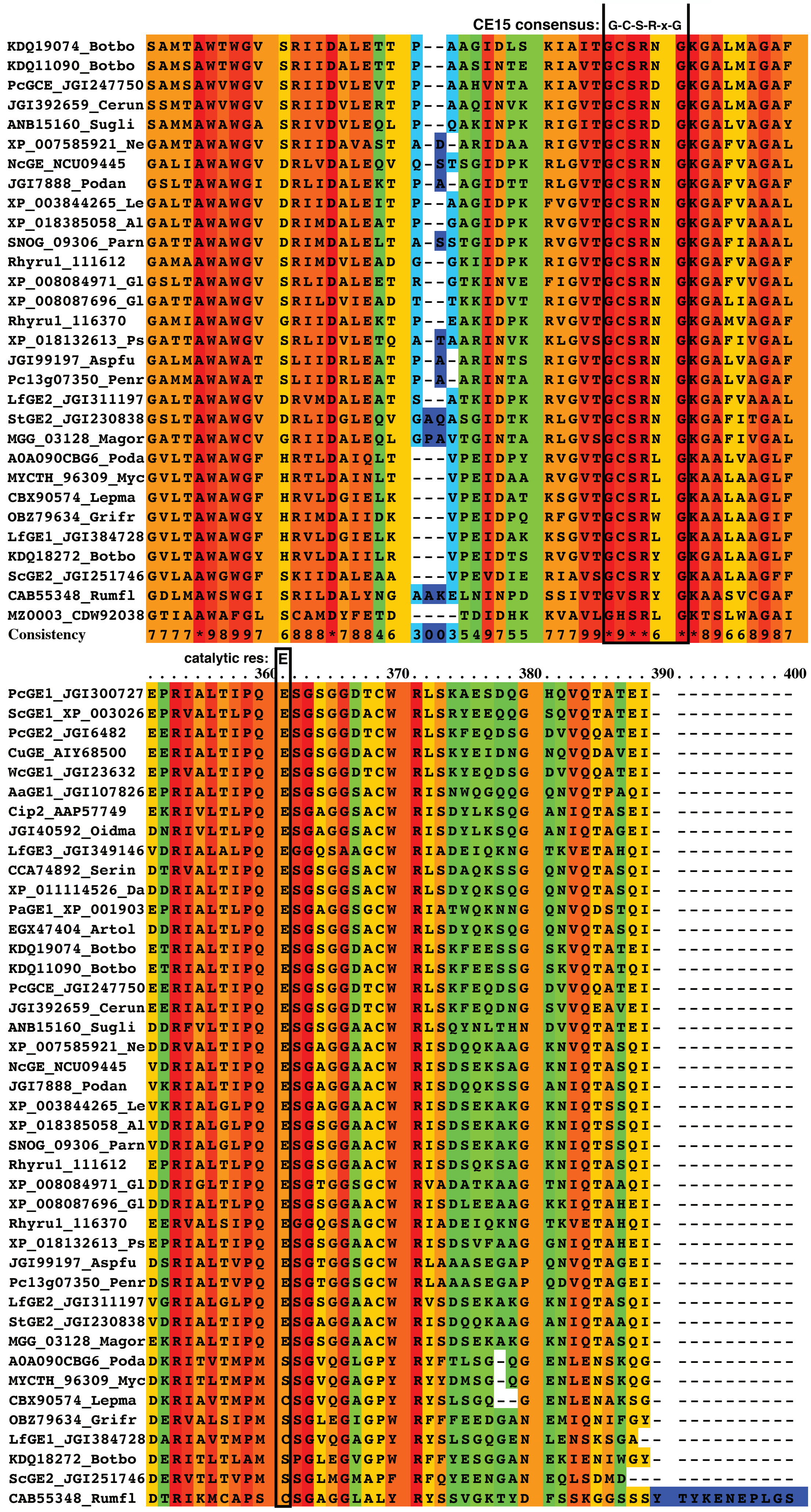

CE15 consensus: G-C-S-R-x-G
KDQ19074_Botbo SAMTAWTWGV SRIIDALETT P--AAGIDLS KIAITGCSRN GKGALMAGAF
KDQ11090_Botbo SAMSAWTWGV SRIIDALETT P--AASINTE RIAVTGCSRN GKGALMIGAF
PcGCE_JGI247750 SAMSAWVWGV SRIIDVLEVT P--AAHVNTA KIAVTGCSRD GKGALMAGAF
JGI392659_Cerun SSMTAWVWGV SRIIDVLERT P--AAQINVK KIGVTGCSRN GKGALMAGAF
ANB15160_Sugli SAMMAWAWGA SRIVDVLEQL P--QAKINPK RIGITGCSRD GKGALVAGAY
XP_007585921_Ne GAMTAWAWGV SRIIDAVAST A-D-ARIDAA RIGVTGCSRN GKGAFVAGAL
NcGE_NCU09445 GALIAWAWGV DRLVDALEQV Q-STSGIDPK RLGVTGCSRN GKGAFVAGAL
JGI7888_Podan GSLTAWAWGI DRLIDALEKT P-A-AGIDTT RLGVTGCSRN GKGAFVAGAF
XP_003844265_Le GALTAWAWGV DRIIDALEIT P--AAGIDPK FVGVTGCSRN GKGAFVAAAL
XP_018385058_Al GALTAWAWGV DRIMDALEAT P--GAGIDPK RVGVTGCSRN GKGAFVAAAL
SNOG_09306_Parn GATTAWAWGV DRIMDALELT A-SSTGIDPK RVGVTGCSRN GKGAFIAAAL
Rhyru1_111612 GAMAAWAWGV SRIMDVLEAD G--GKIIDPK RIGVTGCSRN GKGAFVAGAF
XP_008084971_Gl GSLTAWAWGV SRLIDALEET R--GTKINVE FIGVTGCSRN GKGAFVVGAL
XP_008087696_Gl GATTAWAWGV SRILDVIEAD T--TKKIDVT RIGVTGCSRN GKGALIAGAL
Rhyru1_116370 GAMIAWAWGV GRIIDALEKT P--EAKIDPK RVGVTGCSRN GKGAMVAGAF
XP_018132613_Ps GATTAWAWGV SRLIDVLETQ A-TAARINVK KLGVSGCSRN GKGAFIVGAL
JGI99197_Aspfu GALMAWAWAT SLIIDRLEAT P-A-ARINTS RIGVTGCSRN GKGALVAGAF
Pc13g07350_Penr GAMMAWAWAT SLIIDRLEAT P-A-ARINTA RIGVTGCSRN GKGALVAGAF
LfGE2_JGI311197 GALTAWAWGV DRVMDALEAT S--ATKIDPK RVGVTGCSRN GKGAFVAAAL
StGE2_JGI230838 GSLTAWAWGV DRLIDGLEQV GAQASGIDTK RLGVTGCSRN GKGAFITGAL
MGG_03128_Magor GATTAWAWCV GRIIDALEQL GPAVTGINTA RLGVSGCSRN GKGAFIVGAL
A0A090CBG6_Poda GVLTAWAWGF HRTLDAIQLT ---VPEIDPY RVGVTGCSRL GKAALVAGLF
MYCTH_96309_Myc GVLTAWAWGF HRTLDAINLT ---VPEIDAA RVGVTGCSRL GKAALAAGLF
CBX90574_Lepma GVLTAWAWGF HRVLDGIELK ---VPEIDAT KSGVTGCSRL GKAALAAGLF
OBZ79634_Grifr GVLTAWAWGY HRIMDAIIDK ---VPEIDPQ RFGVTGCSRW GKAALAAGIF
LfGE1_JGI384728 GVLTAWAWGF HRVLDGIELK ---VPEIDAK KSGVTGCSRL GKAALAAGLF
KDQ18272_Botbo GVLTAWAWGY HRVLDAIILR ---VPEIDTS RVGVTGCSRY GKGALAAGLF
ScGE2_JGI251746 GVLAAWGWGF SKIIDALEAA ---VPEVDIE RIAVSGCSRY GKAALAAGFF
CAB55348_Rumfl GDLMAWSWGI SRILDALYNG AAKELNINPD SSIVTGVSRY GKAASVCGAF
MZ0003_CDW92038 GTIAAWAFGL SCAMDYFETD ----TDIDHK KVAVLGHSRL GKTSLWAGAI
Consistency 7777*98997 6888*78846 3003549755 77799*9**6 **89668987
catalytic res: E
.........360.........370.........380.........390.........400
PcGE1_JGI300727 EPRIALTIPQ ESGSGGDTCW RLSKAESDQG HQVQTATEI- ----------
ScGE1_XP_003026 EPRVALTLPQ ESGSGGDACW RLSRYEEQQG SQVQTATEI- ----------
PcGE2_JGI6482 EERIALTIPQ ESGSGGDTCW RLSKFEQDSG DVVQQATEI- ----------
CuGE_AIY68500 EERIALTIPQ ESGSGGDACW RLSKYEIDNG NQVQDAVEI- ----------
WcGE1_JGI23632 EPRVALTIPQ ESGSGGDTCW RLSKYEQDSG DVVQQATEI- ----------
AaGE1_JGI107826 EPRIALTIPQ ESGSGGSACW RISNWQGQQG QNVQTPAQI- ----------
Cip2_AAP57749 EKRIVLTLPQ ESGAGGSACW RISDYLKSQG ANIQTASEI- ----------
JGI40592_Oidma DNRIVLTLPQ ESGAGGSACW RISDYLKSQG ANIQTAGEI- ----------
LfGE3_JGI349146 VDRIALALPQ EGGQSAAGCW RIADEIQKNG TKVETAHQI- ----------
CCA74892_Serin DTRVALTIPQ ESGSGGSACW RLSDAQKSSG QNVQTASQI- ----------
XP_011114526_Da DDRIALTIPQ ESGSGGSACW RLSDYQKSQG QNVQTASQI- ----------
PaGE1_XP_001903 EPRIALTLPQ ESGAGGSGCW RIATWQKNNG QNVQDSTQI- ----------
EGX47404_Artol DDRIALTLPQ ESGSGGSACW RLSDYQKSQG QNVQTASQI- ----------
KDQ19074_Botbo ETRIALTIPQ ESGSGGDACW RLSKFEESSG SKVQTATEI- ----------
KDQ11090_Botbo ETRIALTIPQ ESGSGGDACW RLSKFEESSG SKVQTATQI- ----------
PcGCE_JGI247750 EERIALTIPQ ESGSGGDTCW RLSKFEQDSG DVVQQATEI- ----------
JGI392659_Cerun EERIALTIPQ ESGSGGDTCW RLSKFEQDNG SVVQEAVEI- ----------
ANB15160_Sugli DDRFVLTIPQ ESGSGGAACW RLSQYNLTHN DVVQTATEI- ----------
XP_007585921_Ne DDRVALTIPQ ESGAGGAACW RISDQQKAAG KNVQTASQI- ----------
NcGE_NCU09445 VDRIALTIPQ ESGAGGAACW RISDSEKSAG KNIQTASQI- ----------
JGI7888_Podan VKRIALTIPQ ESGAGGAACW RISDQQKSSG ANIQTASQI- ----------
XP_003844265_Le VKRIALGLPQ ESGAGGAACW RISDSEKAKG KNIQTSSQI- ----------
XP_018385058_Al VDRIALGLPQ ESGSGGAACW RISDSEKAKG KNIQTSSQI- ----------
SNOG_09306_Parn VDRIALGLPQ ESGSGGAACW RISDSEKAKG KNIQTSSQI- ----------
Rhyru1_111612 EPRIALTLPQ ESGSGGAACW RISDSQKSAG KNIQTASQI- ----------
XP_008084971_Gl DDRIGLTIPQ ESGTGGSGCW RVADATKAAG TNIQTAAQI- ----------
XP_008087696_Gl DDRIALTLPQ ESGSGGAACW RISDLEEAAG KKIQTAHEI- ----------
Rhyru1_116370 EERVALSIPQ EGGQGSAGCW RIADEIQKNG TKVETAHQI- ----------
XP_018132613_Ps EPRIALTIPQ ESGSGGAACW RISDSVFAAG GNIQTAHEI- ----------
JGI99197_Aspfu DSRIALTVPQ ESGTGGSGCW RLAAASEGAP QNVQTAGEI- ----------
Pc13g07350_Penr DSRIALTVPQ ESGTGGSGCW RLAAASEGAP QDVQTAGEI- ----------
LfGE2_JGI311197 VGRIALGLPQ ESGSGGAACW RVSDSEKAKG KNIQTASQI- ----------
StGE2_JGI230838 VDRIALTIPQ ESGAGGAACW RISDQQKAAG ANIQTAAQI- ----------
MGG_03128_Magor EKRIALTIPQ ESGSGGAACW RISDSEKAKG KNIQTASQI- ----------
A0A090CBG6_Poda DKRITVTMPM SSGVQGLGPY RYFTLSG-QG ENLENSKQG- ----------
MYCTH_96309_Myc DKRITLTMPM SSGVQGAGPY RYYDMSG-QG ENLENSKQG- ----------
CBX90574_Lepma DKRIAVTMPM CSGVQGAGPY RYSLSGQ--G ENLENAKSG- ----------
OBZ79634_Grifr DERVALSIPM SSGLEGIGPW RFFFEEDGAN EMIQNIFGY- ----------
LfGE1_JGI384728 DARIAVTMPM CSGVQGAGPY RYSLSGQGEN LENSKSGA-- ----------
KDQ18272_Botbo DERITLTLAM SPGLEGVGPW RFFYESGGAN EKIENIWGY- ----------
ScGE2_JGI251746 DERVTLTVPM SSGLMGMAPF RFQYEENGAN EQLSDMD--- ----------
CAB55348_Rumfl DTRIKMCAPS CSGAGGLALY RYSSVGKTYD FSSKGGSSSY TYKENEPLGS

```
MZ0003_CDW92038 DTRFALTISN CSGCGGAALS RRRFGE---- -TVRRINTS- ----------
Consistency     6479797898 79*6795668 *674455447 4588674660 0000000000

                .........410.........420.........430.........440.........450
PcGE1_JGI300727 ---VTENVWF STNFNNYVNN LNVLPYDHHM LMALVA--PR -ALVSFENTD
ScGE1_XP_003026 ---VGENCWF SAGFDQYVNN LDSLPYDHHL LAALVA--PR -GLISYANTD
PcGE2_JGI6482   ---VQENVWF STNFDNYVFN ISLLPYDHHE LAALVA--PR -PLISYENTD
CuGE_AIY68500   ---VGENVWF STNFNNYVQK LPTVPEDHHL LAAMVA--PR -AMISFENTD
WcGE1_JGI23632  ---VTENVWF STNFDNYVNN LSVLPYDHHE LAAMVA--PR -PLLSYENTE
AaGE1_JGI107826 ---ITENVWL GPVFNNHANN VNALPFDHHQ LAGLIA--PR -ALYVIENSD
Cip2_AAP57749   ---IGEDPWF STTFNSYVNQ VPVLPFDHHS LAALIA--PR -GLFVIDNNI
JGI40592_Oidma  ---IGEDPWF SSNFNNYVNQ VPILPFDHHS LAALIA--PR -GLFVIDNNI
LfGE3_JGI349146 ---VNGDSWF STDFSKYVDT VPTLPWDNHM LHALYAYPPR -GLLIIENTA
CCA74892_Serin  ---ITENVWF SKSFDPFVTS VTRLPFDHHL LAGMVA--PR -GLLVIENTS
XP_011114526_Da ---ITENVWF STNFNANVNQ VNRLPFDHHS LAGLVA--PR -GLYVIENTS
PaGE1_XP_001903 ---VQENVWF SPNFNSYVNN VNQLPFDHHL LAGLIA--PR -ALYVMENVD
EGX47404_Artol  ---ITENVWF STAFNANVNQ VNRLPFDHHS LAALVA--PR -GLYVIENTS
KDQ19074_Botbo  ---VGENVWF STNFNNYVNT LGKLPFDHHS LMAMVA--PR -GLISFENTD
KDQ11090_Botbo  ---VTENVWF SSGFNSYVNQ LSKLPYDHHS LVALVA--PR -GLVSFENTD
PcGCE_JGI247750 ---VQENVWF STNFDNFVFN ISVLPYDHHS LAGLIA--PR -PMISYENTD
JGI392659_Cerun ---VQEDVWF STNFENFVHN ISVLPYDHHF LAGMVA--PR -PMISFENTD
ANB15160_Sugli  ---VQENVWF STSFERYANY TDQLPLDHHI LAGLVA--PR -GLAIFENIG
XP_007585921_Ne ---VTENVWF SKAFEQYTSR VPSLPEDHHM LAALVA--PR -GLLVIEND-
NcGE_NCU09445   ---VTENVWF SPAFNAYTRQ TTNIPADHHM LAALTV--PR -GLIAFEND-
JGI7888_Podan   ---IGEQPWF SKNFDAHVRS ITNIPQDHHF LAAMIV--PR -GLAVFENN-
XP_003844265_Le ---VGENVWF STRFNAFSTK ASTTSTDHHQ LAGMVA--PR -GLLVIENE-
XP_018385058_Al ---VGENVWF STRFNSFSTK SNTLATDHHQ LAGMVA--PR -GLLVIENE-
SNOG_09306_Parn ---VTENVWF STRFNAFSTK ANTLATDHHQ LAGMVA--PR -GLLVIENE-
Rhyru1_111612   ---VGENVWF SPNFNSFSTK TNTLATDHHE LAALVA--PR -GLFVIEND-
XP_008084971_Gl ---ITENTWL SQRFDPWTTK VSSLPIDHHL LAGLVA--PR -GLLVIDND-
XP_008087696_Gl ---IGENVWY SKRFDPFATK TSTLAVDHHM LPALIA--PR -GLLVIENN-
Rhyru1_116370   ---VNGDTWY STTFPKYVDT VPTIPFDNHL LHALYAYPAR -GLLIIENTA
XP_018132613_Ps ---VGENVWF SPNFNAYVNT IPTMPFDHHM LAGLVA--PR -GLFVIENNI
JGI99197_Aspfu  ---VQENVWF STAFNAYAND EDRLPFDHHM LAGLIA--PR -GLLSIDNAG
Pc13g07350_Penr ---VQENVWF SAAFNTYAND VDRPPFDHHM LAGLIA--PR -GLVSIDNAG
LfGE2_JGI311197 ---VGENVWF SKNFNAFSTK ANTLATDHHQ LAGMVA--PR -GLLVIENE-
StGE2_JGI230838 ---ITENPWF SRNFDPHVNS ITSVPQDHHL LAALIV--PR -GLAVFENN-
MGG_03128_Magor ---VTENAWF SPRFNSFTSR TTTIPADHHF LAAMVA--PR -GLFVIEND-
A0A090CBG6_Poda ---A--GWWS NSKLGSFMNH HENLPFDAHT IAAAIA--PR -ALIIDQGTG
MYCTH_96309_Myc ---A--GWWT NSKLGTFVNH AQNLPYDAHT IVAAIA--PR -AVIIDQGTG
CBX90574_Lepma  ---A--GWWT SSGISQFVGK STQLPYDAHT IVAAIA--PR -AVILSQNSG
OBZ79634_Grifr  ---A--PYWS TSRLGQFVPN VTNLPFDAHL HAALVA--PR -AIVWDEGEI
LfGE1_JGI384728 ------GWWT SSGISQFVGK STQLPYDAHT IVAAIA--PR -AVILEQGAS
KDQ18272_Botbo  ---A--PWWV STRIGEFVND ARKLPFDAHS IAAVIA--PR -ALIWDVGTS
ScGE2_JGI251746 ---DTSSSWP DDTLVSFRND PARLPVDSNF ITAGVAPR-- -ALIWDEGTT
CAB55348_Rumfl  LQASGEQGWF NGRFMEFR-N AEQFPMDQHM LGALCCDPDR YLFIIGSCES
MZ0003_CDW92038 -----FPHWF CSRFHQYNDK EDKLPIDQHM LIALCA--PR -PVLINSATE
Consistency     00073664*7 8548546655 544884*694 8777890089 0686547752

                                                           catalytic res: H
                .........460.........470.........480.........490.........500
PcGE1_JGI300727 YTWLSPMSAW G-CVNAAHTV FSALGV---- ------ADHH GFAQVGGHAH
ScGE1_XP_003026 YVWLSGMSSF G-CMTAAHAV YEALGV---- ------PENH GFEQVGGHSH
PcGE2_JGI6482   FEWLSPLSGF G-CMTAAHTV WEAMGI---- ------PDKH GFVQVGNHSH
CuGE_AIY68500   YLWLSPMSSF G-CMTAAHTV WQGLGI---- ------ADSH GFAQVGGHAH
WcGE1_JGI23632  YEWLSPLSAY G-CMSAAHTV YEALGI---- ------PDYH GFVQVGNHSH
AaGE1_JGI107826 MEWLGWTATY G-CMAAARTQ WEALGA---- ------LDNF GFSQVGGNQH
Cip2_AAP57749   D-WLGPQSCF G-CMTAAHMA WQALGV---- ------SDHM GYSQIGAHAH
JGI40592_Oidma  D-WLGPQSCF G-CMTTARMA YQALGV---- ------TNNM GYSQIGAHAH
LfGE3_JGI349146 IDYLGPTSNY H-CATAGRKV HEALGV---- ------KDYF GFSQNSHSDH
CCA74892_Serin  MEWLGNMSCF G-CMKAGQTI YQALGV---- ------QSNM GFSQVGNHNH
XP_011114526_Da MEWLGALSTY G-CMKVGSLI YQALGV---- ------SSNM GYSQVGNHNH
PaGE1_XP_001903 MEWLGKISTY G-CMGIARKQ WEALGA---- ------LDNF GYSQVGGNSH
EGX47404_Artol  MEWLGALSTY G-CMKAGSLV YQALGV---- ------SSNM GYSQVGHGDH
KDQ19074_Botbo  FVWLSPMSSW G-CASAAHTV YKALGV---- ------PQNH GFAQIGGHNH
KDQ11090_Botbo  FIWLSPMSNW G-CVSAGRTV YRALGV---- ------PQNH GFAQVGGHNH
PcGCE_JGI247750 FEWLSPLSGF G-CMTAAHPI WEAMGV---- ------PDNH GFVQVGNHSH
JGI392659_Cerun LVWLSPMSGF G-CMTAAHSV WQALGV---- ------PDNH GFVQVGGHAH
ANB15160_Sugli  YPWLGPWSVY G-CMVAARNI YEAVGA---- ------TSNF GFSQDGPHNH
XP_007585921_Ne IDWLGPVSTT G-CMKVGRLI YKAAGA---- ------PDAM GFSLVGGHNH
NcGE_NCU09445   IDWLGPVSTT A-CMQAGRLI YKAYGV---- ------SNHM GFSLVGGHGH
JGI7888_Podan   IDWLGPVSTT G-CMRAGRQI YKAYGV---- ------PNNM GFSLIGGHNH
XP_003844265_Le IDWLGPVSTT A-CMKAGRLI YKALGV---- ------PDNM GLTGSGNHNH
XP_018385058_Al IDWLGPVSTT A-CMKAGRLI YKALGV---- ------PDNM GFTGSGNHNH
SNOG_09306_Parn IDWLGPGSTT A-CMKAGRLI YKAYGV---- ------PANM GFTGSGNHNH
Rhyru1_111612   IDWLGPVSTT G-CMKVGRLI YKAVGA---- ------ADAM GFGLVGGHSH
```

```
XP_008084971_Gl IDWLGPTSTA V-CMGVGQLV YKALGE---- ------TDAF GYSDVGAHNH
XP_008087696_Gl IDWLGPVSTT G-CMRVGALA YQALGA---- ------TDSF GFSEVAPHNH
Rhyru1_116370   IDYLGPPSNY M-CAKAGRMV FQSLGI---- ------TDYM GFSQNSHSNH
XP_018132613_Ps D-WLGPMATT G-CMKTGQMI YKALGV---- ------PDNM GFSETTPHSH
JGI99197_Aspfu  YQWLGPCSSL G-CMGTARLV WQAMGV---- ------PDRM GYSMSTNHPH
Pc13g07350_Penr YQWLGPWSSL G-CMGTARLI WQAMGV---- ------PDRM GYSMSTDHPH
LfGE2_JGI311197 IDWLGPVSTT A-CMKAGRLI YKALGV---- ------PDNM GFTGSGNHAH
StGE2_JGI230838 IDWLGPVSTT G-CMAAGRLI YKAYGV---- ------PNNM GFSLVGGHNH
MGG_03128_Magor IDWLGPVSTT A-CMKAGRAI YQALGV---- ------GPSM GFSLVGGHQH
A0A090CBG6_Poda DQFVNSKGTA TVIFPAAKVV YDWLGA---- ------GDKI GINVRSGG-H
MYCTH_96309_Myc DPFVNSKGTA VVVYPAAKVV YDWLGA---- ------GENI GISVRGGG-H
CBX90574_Lepma  DQFTDSKGTA QITFPAAKVV YKWLGV---- ------GEKL GMSIPSGG-H
OBZ79634_Grifr  DYWTNPEGEA SVTFPAAHAV YEWLGA---- ------GDRV GVAIRPGD-H
LfGE1_JGI384728 DQFTDSKGTA TVTFPAAKAV YNWLGV---- ------GPQL GMAIPKGG-H
KDQ18272_Botbo  DYWTNPEGES AVTFPAAKAV YNWLGA---- ------SGQV GLATRSGG-H
ScGE2_JGI251746 DYWTNPEGTA AVTFPATLAL YEWLGA---- ------GDNV GIALRNSG-H
CAB55348_Rumfl  EDWVNAPSVW MAY-LGMKHV WDYVGI---- ------SDHL AINIHKSG-H
MZ0003_CDW92038 DKWADPHGEF L-AAQGADAV YRMLGTGGLD AKKWPEPNKL VKSTIGYHLR
Consistency     4487664854 5076476547 7667*70000 0000004655 9664465639

                .........510.........520.........530.........540.........550
PcGE1_JGI300727 CAWP---DSL TPSLNAFFNR FLLDQ-NV-- DTNVFTTNNQ F------GGA
ScGE1_XP_003026 CQWP---SQL DGSLNAFINK FLLGQ-DV-- STDYFESNNQ F------NGV
PcGE2_JGI6482   CDFP---SSL NPTLFAFFDK FLLGK-EA-- NTSIFETNGL F------NGT
CuGE_AIY68500   CAWP---SSL TPQLNAFINR FLLDQ-SA-- TTNVFTTNNQ F------GKV
WcGE1_JGI23632  CYFP---DTL DDSLYAFFDR FLLDEDDV-- STDYFTTNYQ F------NGT
AaGE1_JGI107826 CSFN--SGKQ SAELNAFINK FLLQSGGG-- TTSILRTERN H------G--
Cip2_AAP57749   CAFP---SNQ QSQLTAFVQK FLLGQ-ST-- NTAIFQSD-F S---------
JGI40592_Oidma  CSFP---SNQ QSQLNAFVQQ FLLGQ-SA-- NTNIFQSD-F T---------
LfGE3_JGI349146 CGFP---KAQ QPELTAFIER FLLAK-DT-- KTDVWKTD-G K------F--
CCA74892_Serin  CSFP---SSQ QTELTAFVQR FLFDQSSA-- NTAIFKTD-G N------L--
XP_011114526_Da CAFP---SSQ QSELNAFANR FLLDQ-SV-- ATNIFRTD-G S------F--
PaGE1_XP_001903 CSFP--SSQQ GSELNAFIEK FLLKRSGG-- NTNIFRSTQT H------S--
EGX47404_Artol  CGFP--AAQQ GSELNSFINR FLLDQ-SV-- TTNVFRTD-G N------F--
KDQ19074_Botbo  CAWP---TSL TPTLNAFFDK FLLGKSVN-- TDYFTTNSVF G------PA-
KDQ11090_Botbo  CAWP---SSL TPTLNAFFDK FLFGKNVN-- TDFFSTNNVF G------PA-
PcGCE_JGI247750 CEFP---SDL NPTLFAFFDK FLLGKEAN-- TTIFETNEVF N------GT-
JGI392659_Cerun CAFP---STL NDQLFSFIDR FLLNEPAN-- TTVFTTNDVF N------NV-
ANB15160_Sugli  CSFP--TDDQ GAELQAFYNK FLLDENVN-- TNVFRTN--- G------GY-
XP_007585921_Ne CSFP---SAQ QPELSAFIDK YLLKGSSS-- TANIEKSN-- Q---------
NcGE_NCU09445   CQFP---SSQ QSELTSYINY FLLKAGTA-- PGAVERSS-- A---------
JGI7888_Podan   CQFP---SGQ NSELNQYINY FLLKSGTA-- PGSVERST-- S---------
XP_003844265_Le CSFP---SNQ QTELTAFINK FLLGQSSN-- TANIEKGP-- S---------
XP_018385058_Al CQFP---SNQ QADLTAFINK FLLGQSSN-- TANIEKGP-- S---------
SNOG_09306_Parn CSFP---SNQ GADLTAFINK FLLNQ-SA-- ETNIEKGP-- A---------
Rhyru1_111612   CQFP---SSQ QADLTAFFNK FLLGG-TG-- NTDVEKSP-- A---------
XP_008084971_Gl CLFP---DSQ KPQLEAFVKK WLFDKEVD-- TDGVFVTS-- R---------
XP_008087696_Gl CTFP---SSQ QTELTAFINR FLKGDASA-- KTAGVDKS-- D------QA-
Rhyru1_116370   CGFP---SAQ QPELTAFINR FLLNQ-TA-- DTNVFKTD-- G------KF-
XP_018132613_Ps CSFP---SSQ QADLTAFVQK FLFDQ-NT-- NTAVSKSD-- Q---------
JGI99197_Aspfu  CSFP---DQQ REDLFAFINR FLLGM-EV-- NTTVQKNY-- A------GI-
Pc13g07350_Penr CSFP---DQQ RGDLFAFINR FMLGM-EV-- NTTVQKNY-- A------GI-
LfGE2_JGI311197 CQFP---SNQ QSDLTAYINK FLLNQDAN-- TANIEKGP-- ----------
StGE2_JGI230838 CQFP---SSQ NQDLNSYINY FLLGQGSP-- SGVEHSDV-- ----------
MGG_03128_Magor CQFP---SAT TANLNSYINR FLLGTGQT-- NNVETSRA-- ----------
A0A090CBG6_Poda CD-----MSG FNGILPFVQK IFFNTTTTRN FHSLGTYN-T P------LA-
MYCTH_96309_Myc CD-----LSG YTAILPYVQK IFFGTPTDKD YNNLGSYG-S P------VS-
CBX90574_Lepma  CD-----QNG YADVLPFVQK VLQGKSTTRD YDDLKTWKAM P------EAY
OBZ79634_Grifr  CD-----ISG YTNIQDFMNK IFFNEKITRN YSDISPY--T P------YTE
LfGE1_JGI384728 CD-----MSG YADVLPFVQK ILQGKTTTRN YDDLGSW--K A------MP-
KDQ18272_Botbo  CD-----NAG FTNVQGFMNK IFFGKTPSKN YDSTSPY--T A------HT-
ScGE2_JGI251746 CDP----S-G NTNVADFINK VFFGTET--- DRDYHDI--- --------SP
CAB55348_Rumfl  AVI----AED IEKMVQYFDY HVYGIQP--- KMNLEELQTS VFALPKNKDS
MZ0003_CDW92038 PGKHDVTARD WDVYIEFADH HMTGGAE--- ---------- ----------
Consistency     9455000655 4458469667 7865514300 4545344301 2000000100

                .........560.........570.........580.........590.
PcGE1_JGI300727 TWTQSSWI-N W----STPTL S--------- ---------- -
ScGE1_XP_003026 TWSESQWI-N W----ETPTL N--------- ---------- -
PcGE2_JGI6482   EWVASQWI-N W----TTPRF T--------- ---------- -
CuGE_AIY68500   QWNAANWI-T W----TTPTL T--------- ---------- -
WcGE1_JGI23632  VWNASYWI-N W----TTPQL D--------- ---------- -
AaGE1_JGI107826 SFNLAEWT-P W----NVPNL R--------- ---------- -
Cip2_AAP57749   -ANQSQWI-D W----TTPTL S--------- ---------- -
JGI40592_Oidma  -FSQSQWI-D W----TVPTL T--------- ---------- -
```

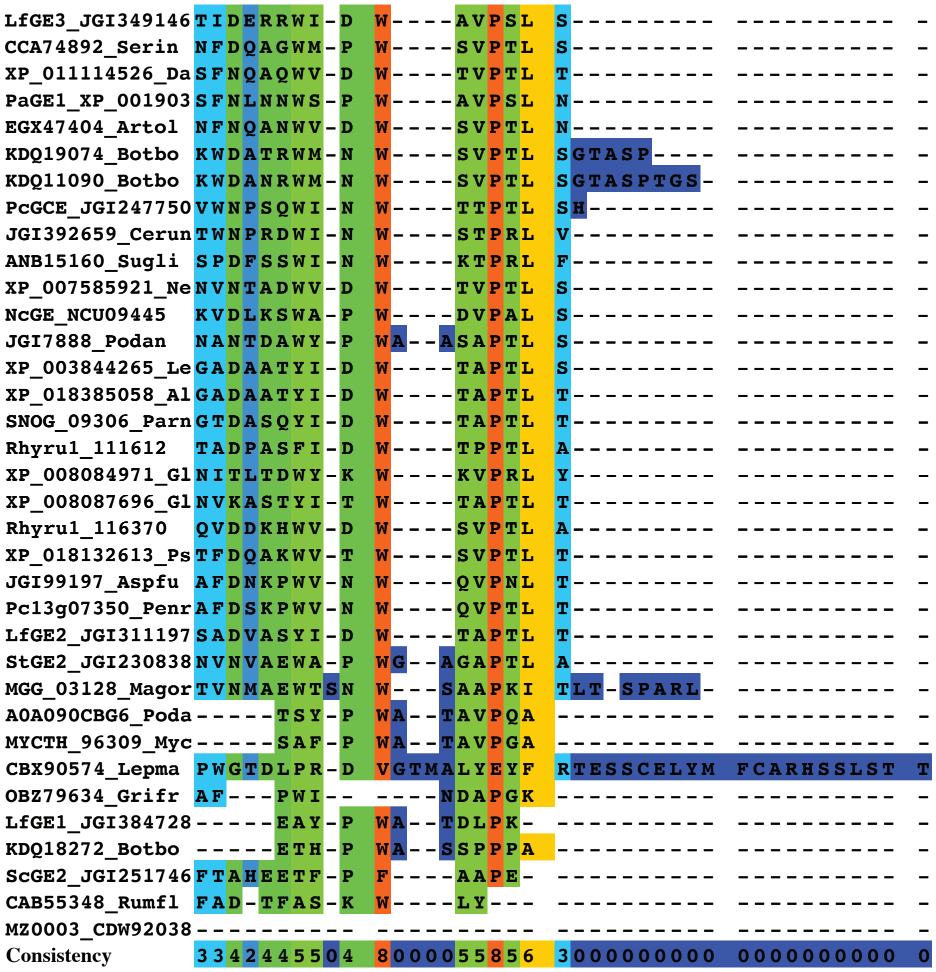

Figure S4. Multiple sequence alignment of CE15 domain containing proteins. The conservation scoring was performed by PRALINE (http://www.ibi.vu.nl/programs/PRALINEwww/; Simossis & Heringa, 2005). The scoring scheme works from 0 (blue) for the least conserved alignment position, up to 10 for the most conserved alignment position (red). The conserved CE15 consensus motif G-C-S-R-x-G, containing the catalytic serine, as well as the catalytic histidine and aspartic acid residues are labelled.

References

Berka RM, Grigoriev I V, Otillar R, Salamov A, Grimwood J, Reid I, Ishmael N, John T, Darmond C, Moisan M-C, Henrissat B, Coutinho PM, Lombard V, Natvig DO, Lindquist E, Schmutz J, Lucas S, Harris P, Powlowski J, Bellemare A, Taylor D, Butler G, de Vries RP, Allijn IE, van den Brink J, Ushinsky S, Storms R, Powell AJ, Paulsen IT, Elbourne LDH, Baker SE, Magnuson J, LaBoissiere S, Clutterbuck AJ, Martinez D, Wogulis M, de Leon AL, Rey MW, Tsang A (2011) Comparative genomic analysis of the thermophilic biomass-degrading fungi *Myceliophthora thermophila* and *Thielavia terrestris*. Nat Biotech 29:922–927.

Biely P, Malovíková A, Uhliariková I, Li XL, Wong DWS (2015) Glucuronoyl esterases are active on the polymeric substrate methyl esterified glucuronoxylan. FEBS Lett 589:2334–2339. doi: 10.1016/j.febslet.2015.07.019

D'Errico C, Jørgensen JO, Krogh KBRM, Spodsberg N, Madsen R, Monrad RN (2015) Enzymatic degradation of lignin-carbohydrate complexes (LCCs): Model studies using a fungal glucuronoyl esterase from Cerrena unicolor. Biotechnol Bioeng 112:914–922. doi: 10.1002/bit.25508

De Santi C, Willassen NP, Williamson A (2016) Biochemical Characterization of a Family 15 Carbohydrate Esterase from a Bacterial Marine Arctic Metagenome. PLoS One 11:e0159345. doi: 10.1371/journal.pone.0159345

Ďuranová M, Hirsch J, Kolenová K, Biely P (2009a) Fungal Glucuronoyl Esterases and Substrate Uronic Acid Recognition. Biosci Biotechnol Biochem 73:2483–2487. doi: 10.1271/bbb.90486

Ďuranová M, Špániková S, Wösten HAB, Biely P, de Vries RP (2009b) Two glucuronoyl esterases of Phanerochaete chrysosporium. Arch Microbiol 191:133–40. doi: 10.1007/s00203-008-0434-y

Espagne E, Lespinet O, Malagnac F, Da Silva C, Jaillon O, Porcel BM, Couloux A, Aury J-M, Ségurens B, Poulain J, Anthouard V, Grossetete S, Khalili H, Coppin E, Déquard-Chablat M, Picard M, Contamine V, Arnaise S, Bourdais A, Berteaux-Lecellier V, Gautheret D, de Vries RP, Battaglia E, Coutinho PM, Danchin EGJ, Henrissat B, Khoury REL, Sainsard-Chanet A, Boivin A, Pinan-Lucarré B, Sellem CH, Debuchy R, Wincker P, Weissenbach J, Silar P (2008) The genome sequence of the model ascomycete fungus *Podospora anserina*. Genome Biol 9:R77–R77. doi: 10.1186/gb-2008-9-5-r77

Floudas D, Binder M, Riley R, Barry K, Blanchette RA, Henrissat B, Martínez AT, Otillar R, Joseph W. Spatafora, 6 Jagjit S. Yadav, 7 Andrea Aerts, 2 Isabelle Benoit, 8, 9 Alex Boyd, 6 Alexis Carlson, 1 Alex 2* David S. Hibbett1* (2012) The Paleozoic Origin of Enzymatic from 31 Fungal Genomes. Science (80-) 336:1715–1719.

Huynh HH, Arioka M (2016) Functional expression and characterization of a glucuronoyl esterase from the fungus Neurospora crassa: identification of novel consensus sequences containing the catalytic triad. J Gen Appl Microbiol 1–8. doi: 10.2323/jgam.2016.03.004

Katsimpouras C, Bénarouche A, Navarro D, Karpusas M, Dimarogona M, Berrin J-G, Christakopoulos P, Topakas E (2014) Enzymatic synthesis of model substrates recognized by glucuronoyl esterases from Podospora anserina and Myceliophthora

thermophila. Appl Microbiol Biotechnol 98:5507–16. doi: 10.1007/s00253-014-5542-9
Kohler A, Kuo A, Nagy LG, Morin E, Barry KW, Buscot F, Canbäck B, Choi C, Cichocki N, Clum A, Colpaert J, Copeland A, Costa MD, Doré J, Floudas D, Gay G, Girlanda M, Henrissat B, Herrmann S, Hess J, Högberg N, Johansson T, Khouja H-R, LaButti K, Lahrmann U, Levasseur A, Lindquist EA, Lipzen A, Marmeisse R, Martino E, Murat C, Ngan CY, Nehls U, Plett JM, Pringle A, Ohm RA, Perotto S, Peter M, Riley R, Rineau F, Ruytinx J, Salamov A, Shah F, Sun H, Tarkka M, Tritt A, Veneault-Fourrey C, Zuccaro A, Tunlid A, Grigoriev I V, Hibbett DS, Martin F (2015) Convergent losses of decay mechanisms and rapid turnover of symbiosis genes in mycorrhizal mutualists. Nat Genet 47:410–5. doi: 10.1038/ng.3223
Li X-L, Spániková S, de Vries RP, Biely P (2007) Identification of genes encoding microbial glucuronoyl esterases. FEBS Lett 581:4029–35. doi: 10.1016/j.febslet.2007.07.041
Martinez D, Larrondo LF, Putnam N, Gelpke MDS, Huang K, Chapman J, Helfenbein KG, Ramaiya P, Detter JC, Larimer F, Coutinho PM, Henrissat B, Berka R, Cullen D, Rokhsar D (2004) Genome sequence of the lignocellulose degrading fungus Phanerochaete chrysosporium strain RP78. Nat Biotechnol 22:695–700. doi: 10.1038/nbt967
Nierman WC, Pain A, Anderson MJ, Wortman JR, Kim HS, Arroyo J, Berriman M, Abe K, Archer DB, Bermejo C, Bennett J, Bowyer P, Chen D, Collins M, Coulsen R, Davies R, Dyer PS, Farman M, Fedorova N, Fedorova N, Feldblyum T V, Fischer R, Fosker N, Fraser A, Garcia JL, Garcia MJ, Goble A, Goldman GH, Gomi K, Griffith-Jones S, Gwilliam R, Haas B, Haas H, Harris D, Horiuchi H, Huang J, Humphray S, Jimenez J, Keller N, Khouri H, Kitamoto K, Kobayashi T, Konzack S, Kulkarni R, Kumagai T, Lafton A, Latge J-P, Li W, Lord A, Lu C, Majoros WH, May GS, Miller BL, Mohamoud Y, Molina M, Monod M, Mouyna I, Mulligan S, Murphy L, O'Neil S, Paulsen I, Penalva MA, Pertea M, Price C, Pritchard BL, Quail MA, Rabbinowitsch E, Rawlins N, Rajandream M-A, Reichard U, Renauld H, Robson GD, de Cordoba SR, Rodriguez-Pena JM, Ronning CM, Rutter S, Salzberg SL, Sanchez M, Sanchez-Ferrero JC, Saunders D, Seeger K, Squares R, Squares S, Takeuchi M, Tekaia F, Turner G, de Aldana CRV, Weidman J, White O, Woodward J, Yu J-H, Fraser C, Galagan JE, Asai K, Machida M, Hall N, Barrell B, Denning DW (2005) Genomic sequence of the pathogenic and allergenic filamentous fungus *Aspergillus fumigatus*. Nature 438:1151–1156.
Ohm R a, de Jong JF, Lugones LG, Aerts A, Kothe E, Stajich JE, de Vries RP, Record E, Levasseur A, Baker SE, Bartholomew K a, Coutinho PM, Erdmann S, Fowler TJ, Gathman AC, Lombard V, Henrissat B, Knabe N, Kües U, Lilly WW, Lindquist E, Lucas S, Magnuson JK, Piumi F, Raudaskoski M, Salamov A, Schmutz J, Schwarze FWMR, vanKuyk P a, Horton JS, Grigoriev I V, Wösten H a B (2010) Genome sequence of the model mushroom Schizophyllum commune. Nat Biotechnol 28:957–963. doi: 10.1038/nbt.1643
Simossis VA, Heringa J (2005) PRALINE: A multiple sequence alignment toolbox that integrates homology-extended and secondary structure information. Nucleic Acids Res 33:289–294. doi: 10.1093/nar/gki390
Topakas E, Moukouli M, Dimarogona M, Vafiadi C, Christakopoulos P (2010) Functional expression of a thermophilic glucuronyl esterase from Sporotrichum thermophile:

identification of the nucleophilic serine. Appl Microbiol Biotechnol 87:1765–72. doi: 10.1007/s00253-010-2655-7

Tsai AY-L, Canam T, Gorzsás A, Mellerowicz EJ, Campbell MM, Master ER (2012) Constitutive expression of a fungal glucuronoyl esterase in Arabidopsis reveals altered cell wall composition and structure. Plant Biotechnol J 10:1077–87. doi: 10.1111/j.1467-7652.2012.00735.x

Wong DWS, Chan VJ, McCormack A a, Hirsch J, Biely P (2012) Functional Cloning and Expression of the Schizophyllum commune Glucuronoyl Esterase Gene and Characterization of the Recombinant Enzyme. Biotechnol Res Int 2012:951267. doi: 10.1155/2012/951267

Wiśniewski JR, Zougman A, Nagaraj N, Mann M (2009) Universal sample preparation method for proteome analysis. Nat Methods 6:359–62. doi: 10.1038/nmeth.1322
